# Anthraquinone Derivatives from a Marine-Derived Fungus *Sporendonema casei* HDN16-802

**DOI:** 10.3390/md17060334

**Published:** 2019-06-04

**Authors:** Xueping Ge, Chunxiao Sun, Yanyan Feng, Lingzhi Wang, Jixing Peng, Qian Che, Qianqun Gu, Tianjiao Zhu, Dehai Li, Guojian Zhang

**Affiliations:** 1 Key Laboratory of Marine Drugs, Chinese Ministry of Education, School of Medicine and Pharmacy, Ocean University of China, Qingdao 266003, China; 15610568273@163.com (X.G.); sunchunxiao93@163.com (C.S.); yy15715321143@163.com (Y.F.); wlz8866wlz@163.com (L.W.); cheqian064@ouc.edu.cn (Q.C.); guqianq@ouc.edu.cn (Q.G.); zhutj@ouc.edu.cn (T.Z.); dehaili@ouc.edu.cn (D.L.); 2 Laboratory for Marine Drugs and Bioproducts of Qingdao National Laboratory for Marine Science and Technology, Qingdao 266237, China; 3 Key Laboratory of Testing and Evaluation for Aquatic Product Safety and Quality, Ministry of Agriculture and Rural Affairs, Yellow Sea Fisheries Research Institute, Chinese Academy of Fishery Sciences, Qingdao 266071, China; pengjixing1987@163.com

**Keywords:** anthraquinone derivatives, *Sporendonema casei*, marine-derived fungus, cytotoxic activities, antibacterial activities

## Abstract

Five new anthraquinone derivatives, auxarthrols D–H (**1**–**5**), along with two known analogues (**6**–**7**), were obtained from the culture of the marine-derived fungus *Sporendonema casei*. Their structures, including absolute configurations, were established on the basis of NMR, HRESIMS, and circular dichroism (CD) spectroscopic techniques. Among them, compound **4** represents the second isolated anthraquinone derivative with a chlorine atom, which, with compound **6**, are the first reported anthraquinone derivatives with anticoagulant activity. Compounds **1** and **3** showed cytotoxic activities with IC_50_ values from 4.5 μM to 22.9 μM, while compounds **1**, **3**–**4**, and **6**–**7** showed promising antibacterial activities with MIC values from 12.5 μM to 200 μM. In addition, compound **7** was discovered to display potential antitubercular activity for the first time.

## 1. Introduction

Anthraquinones and their derivatives are a group of pigmented polyketides widely produced by fungi. Apart from their bright color attributed to the typical conjugate system in their structure, they have also attracted the attention of scientists due to their diversity of structures and wide range of pharmacological effects, such as their anti-infective, anti-inflammatory, and α-glucosidase inhibitory activities and cytotoxicity against cancer cells [1,2]. Following the discovery of altersolanol A reported in 1967 [3], a series of anthraquinone derivatives have been discovered from various fungal genera, including *Alternaria* [4,5], *Streptomyces* [6,7], *Dactylaria* [8], *Bostryconema* [9], *Stemphylium* [10], *Pleospora* [11], *Auxarthron* [12], *Ampelomyces* [13], *Nigrospora* [14], and *Phomopsis* [15].

During our exploration of novel bioactive secondary metabolites obtained from marine-derived microorganisms, a fungus *Sporendonema casei* HDN16-802 isolated from a sediment sample collected from Zhangzi Island was selected due to its special morphological characteristic (orange color) and tremendous metabolic profile identified via HPLC-UV. Further chemical study generated five new anthraquinones, named auxarthrols D–H (**1**–**5**), along with two known analogues (**6**–**7**). To the best of our knowledge, this is the first time that anthraquinone derivatives have been isolated from the fungus *S. casei*. The cytotoxicity, antibacterial, anticoagulant, and antitubercular activities of **1**–**7** were tested. Herein, we will describe the isolation, structural elucidation, and biological activities of the isolated compounds.

## 2. Results and Discussion

**Figure 1:**
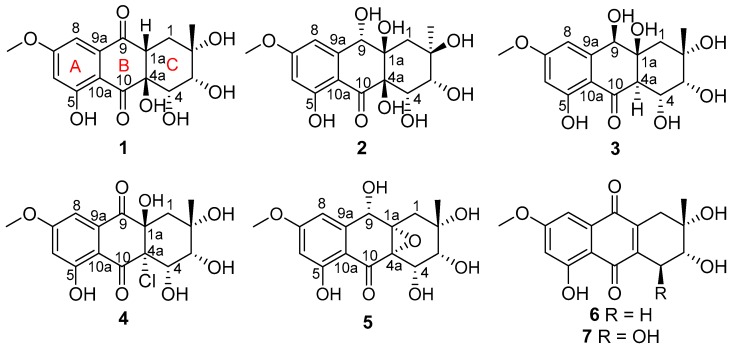
Structures of **1–7**.

*Sporendonema casei* HDN16-802 was cultured (45 L) under static conditions with oatmeal medium at room temperature for one month. The fermentation product (mycelium and broth) was extracted with ethyl acetate to provide the crude extract (10 g). The crude extract was fractionated by different kinds of chromatography, including silica gel vacuum liquid chromatography (VLC), C-18 column chromatography (ODS), Sephadex LH-20 column chromatography, medium performance liquid chromatography (MPLC), and finally HPLC to yield **1** (10.0 mg), **2** (2.1 mg), **3** (5.1 mg), **4** (5.0 mg), **5** (4.5 mg), **6** (10.1 mg), and **7** (5.0 mg) (Figure 1).

**Figure 2:**
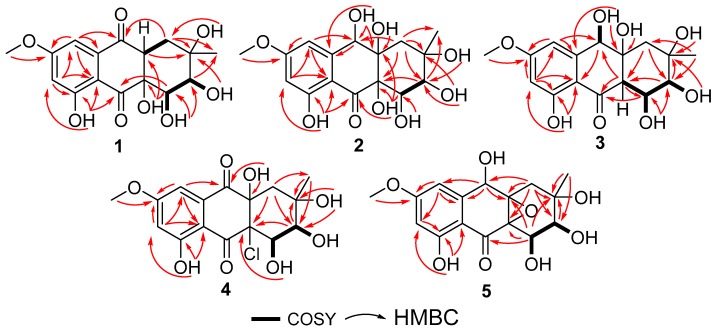
Key HMBC and ^1^H-^1^H COSY correlations of **1**–**5**.

Compound **1** was isolated as a pale yellow solid with the molecular formula C_16_H_18_O_8_, which was established on the basis of the (+)–HRESIMS ion peak at *m*/*z* 339.1076 [M+H]^+^ and *m*/*z* 361.0897 [M+Na]^+^, indicating eight degrees of unsaturation. The 1D NMR (^1^H-NMR, ^13^C NMR, and DEPT) spectrum (Table 1 and Table 2 and Supplementary Materials), together with HSQC correlations (Figure S5), provided five hydroxyl protons, including a chelated hydroxyl at *δ*_H_ 12.44 (s); two methyls, including one methoxy (*δ*_H_ 3.87, s; *δ*_C_ 56.7); one methylene (*δ*_H,_ 1.86, m; *δ*_C_ 34.2); five methines, including two *meta*-coupled aromatic *sp*^2^ methines [*δ*_H_ 6.79, d (2.4), *δ*_C_ 106.1; *δ*_H_ 6.83, d (2.4), *δ*_C_ 105.0] and two *sp*^3^ oxygenated methines [*δ*_H_ 3.61, dd (5.7, 3.1), *δ*_C_ 73.7; *δ*_H_ 4.22, dd (4.5, 3.1), *δ*_C_ 71.3]; and eight non-protonated carbons, including two conjugated ketones (*δ*_C_ 197.3 and 200.1), four aromatic carbons (*δ*_C_ 166.1, 166.3, 110.1, and 137.6), and two oxygenated quaternary (*δ*_C_ 72.3 and 78.2) carbons. A careful comparison of the above signals with those of the known compound auxarthrol B [12] revealed a very similar hydroanthraquinone skeleton, while the most significant differences were the absence of a hydroxyl group and the appearance of a methine signal [*δ*_H_ 3.36, m; *δ*_C_ 48.0] attributed to C-1a (Table 2). The key HMBC correlations from H-1a to C-9 and C-2 (Figure 2 and Supplementary Materials) further confirmed the planar structure of **1**.

**Figure 3:**
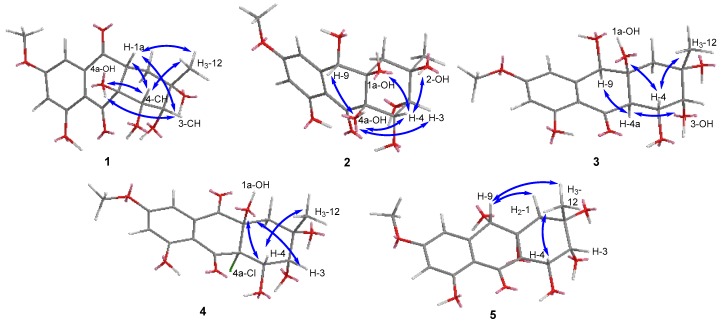
Key NOE correlations of **1–5**.

**Figure 4:**
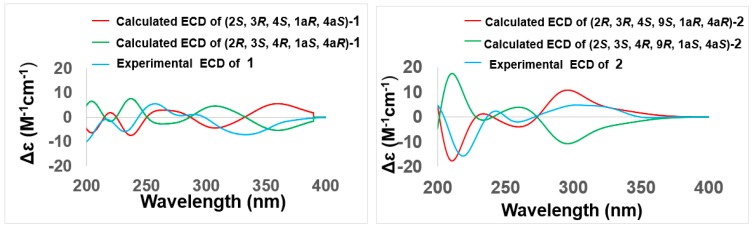
Comparison of the calculated and experimental ECD spectra of **1–2**, auxarthrols D–E.

The relative configuration of the stereogenic carbons in **1** was detected by NOESY correlations and conformational analysis (Figure 3 and Figure S7). The NOESY correlations from H-3 to H-1a and 4a-OH, from H-1a to H-4, and from H-4 to 4a-OH indicated that H-1a and 4a-OH were located on the same face of the molecule, which meant that the B ring and C ring were *cis* fused. The NOESY correlations from H-1a and H-4 to H_3_-12 oriented H_3_-12 to the same side as H-1a and H-4. Computational simulation by Chemdraw (Minimize Energy program), together with the small *J* coupling constant between H-3 and H-4 (^3^*J*_H-3, H-4_ = 3.1 Hz), further confirmed the chair-chair conformation for rings B and C, where H-1a (ax), H-3 (eq), H-4 (ax), 4a-OH (eq), and H_3_-12 (ax) were oriented on the same face, thus completing the relative configuration of the stereogenic carbons in **1** (Figure 3). To determine the absolute configuration of compound **1**, the theoretical calculated electronic circular dichroism (ECD) spectra of possible models were performed using TDDFT. The optimized conformation of the model was obtained and further used for the ECD calculation at the B3LYP/6-31+G(d) level. The pattern (2*S*, 3*R*, 4*S*, 1a*R*, 4a*S*)-**1** of the calculated ECD spectrum was in reasonable agreement with the experimental ECD spectra (Figure 4). Thus, the absolute configuration of **1** was established as 2*S*, 3*R*, 4*S*, 1a*R*, 4a*S*, and we named it auxarthrol D.

Compound **2** was obtained as a pale yellow powder. Its molecular formula of C_16_H_20_O_9_ with seven degrees of unsaturation was determined by the ion peak *m*/*z* 341.1237 [M+H]^+^ in the (+)−HRESIMS. The molecular formula was also corroborated by exploiting ^1^H and ^13^C NMR spectroscopic data (Table 1 and Table 2 and Supplementary Materials). A comparison of these data with **1** revealed the same skeleton with different substitutions. The upfield shift of C-9 (*δ*_C_ 69.0 Vs. *δ*_C_ 197.3) and the downfield shift of C-1a (*δ*_C_ 79.3 Vs. *δ*_C_ 48.0) indicated that both of the C-9 and C-1a positions were substituted by a hydroxyl group in **2** (Table 2). Key HMBC correlations from H-9 to C-9, C-8, and C-1; from 1a-OH to C-1a and C-1; and from 9-OH to C-9 and C-9a (Figure 2 and Supplementary Materials) confirmed the locations of C-9 and C-1a hydroxyl groups, thus completing the planar structure of **2**. The relative configurations of the stereogenic carbons were determined by NOESY correlations and *J* coupling constant analysis (Table 1, Figure 3 and Supplementary Materials). The NOESY correlations from H-4 to both 1a-OH and 4a-OH and from H-3 to 4a-OH, together with the small *J* coupling constant between H-3 and H-4 (^3^*J*_H-3, H-4_ = 3.0 Hz), indicated that 1a-OH, 4a-OH, H-3, and H-4 were located on the same face. Other NOE correlations from H-3 to H_3_-12 and 2-OH and from H-4 to 2-OH with the above evidence suggested a chair conformation of the C ring, where H-3 (eq), H-4 (ax), and 2-OH (ax) were on the same face, while H_3_-12 (eq) was oriented on the opposite face of the molecule. This conformation was further confirmed by using Chemdraw Minimize Energy simulation. Further NOESY correlation from H-9 to 4a-OH indicated that H-9 was on the same face as 4a-OH (Figure 3), thus providing the relative configuration of the stereogenic carbons of **2**. The absolute configuration of **2** was determined by comparing the experimental and calculated ECD spectrum using time-dependent density-functional theory (TDDFT). The good agreement of the calculated ECD spectrum of (2*R*, 3*R*, 4*S*, 9*S*, 1a*R*, 4a*R*)-**2** with that of the experimental spectrum (Figure 4) suggested that the absolute configuration of **2** was 2*R*, 3*R*, 4*S*, 9*S*, 1a*R*, 4a*R*, and we named it auxarthrol E.

**Figure 5:**
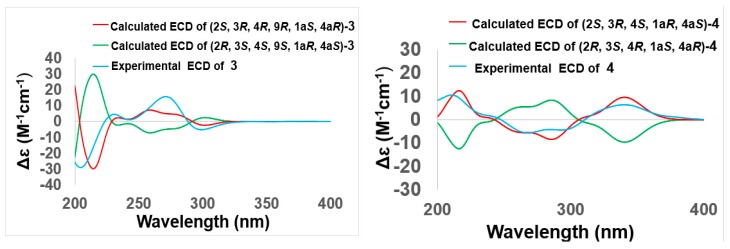
Comparison of the calculated and experimental ECD spectra of **3**–**4**, auxarthrols F–G.

Compound **3** was obtained as a pale yellow powder. The molecular formula of **3** was deduced as C_16_H_20_O_9_ (seven degrees of unsaturation) by (+)–HRESIMS *m/z* 357.1187 [M+H]^+^, which was also corroborated by ^1^H and ^13^C NMR spectroscopic data, as shown in Table 1 and Table 2, which was 16 amu more than the molecular mass of compound **2**, therefore revealing a close relationship between **3** and **2**. According to 1D NMR spectra, the presence of a methine signal at 2.87 ppm and the absence of a hydroxy group in **3** along with the upfield shift of C-4a (*δ*_C_ 53.3 Vs. *δ*_C_ 78.1) suggested that the 4a-OH in **2** was replaced by a hydrogen atom in **3** (Table 1 and Table 2), which was confirmed by the key HMBC correlation from H-4a to C-10 and C-4 (Figure 2 and Supplementary Materials). The relative configurations of the stereogenic carbons were also determined by NOESY correlations and *J* coupling constant analysis. The NOESY correlations from H-9 to H-4a and the large *J* coupling constant between H-4 and H-4a (^3^*J*_H-4, H-4a_ = 9.7 Hz) indicated that H-4 (ax) and 1a-OH (ax) were on the same face, while H-4a (ax) was located on the opposite face, indicating that the B ring and C ring were *trans* fused. By using the Minimize Energy simulation programe in Chemdraw, both B and C rings were proposed to adopt a chair conformation, which provided the lowest steric energy. The NOESY correlation from H-4 to H_3_-12 with the small *J* coupling constant between H-3 and H-4 (^3^*J*_H-3, H-4_ = 3.0 Hz) assigned H_3_-12 and H-3 (eq) on the same face as H-4 (ax) (Figure 3), thus providing the relative configuration of the stereogenic carbons of **3.** The good agreement of the calculated ECD spectrum of (2*S*, 3*R*, 4*R*, 9*R*, 1a*S*, 4a*R*)-**3** with that of the experimental spectrum (Figure 5) suggested that the absolute configuration of **3** was 2*S*, 3*R*, 4*R*, 9*R*, 1a*S*, 4a*R*, and we named it auxarthrol F.

Compound **4** was obtained as a pale yellow powder. Its molecular formula of C_16_H_18_ClO_8_ (eight degrees of unsaturation) was determined by (+)–HRESIMS. The molecular formula was also corroborated by ^1^H and ^13^C NMR spectroscopic data (Table 1 and Table 2), suggesting that the structure of **4** resembled that of paradictyoarthrin A (8) [16], except for the absence of the hydroxyl group on C-9 and the presence of a keto-carbonyl signal at *δ*_C_ 190.6, indicating that the C-9 hydroxyl was replaced by a ketone. Further 2D NMR data confirmed the planar structure of **4** (Figure 2 and Supplementary Materials). The relative configurations of the stereogenic carbons of **4** were established by NOESY correlations and *J* coupling constant analysis (Table 1, Figure 3 and Supplementary Materials). The NOESY correlations from H-4 and H-3 to 1a-OH indicated that 1a-OH and 4a-Cl were located on the opposite side of the B ring, while the NOESY correlation from H-4 to H_3_-12 with a very small *J* coupling constant between H-3 and H-4 suggested that H-3 (eq), H-4 (ax), H_3_-12 (ax), and 1a-OH (ax) were oriented on the same side of the C ring. Moreover, the calculated ECD spectrum of the model compound (2*S*, 3*R*, 4*S*, 1a*R*, 4a*S*)-**4** was well-matched with the experimental ECD spectrum of **4** (Figure 5), thus confirming the absolute structure of **4,** and we named it auxarthrol G.

**Figure 6:**
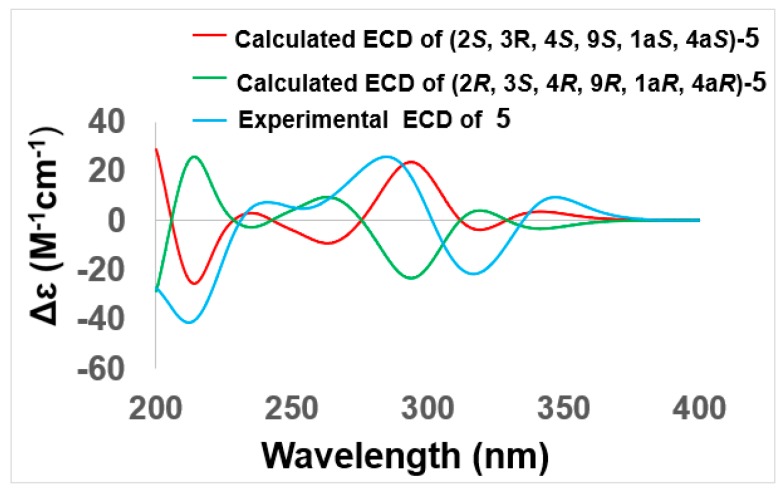
Comparison of the calculated and experimental ECD spectra of **5**, auxarthrol H.

Compound **5** was obtained as a pale yellow solid, with the molecular formula C_16_H_18_O_8_ (eight degrees of unsaturation) from (+)–HRESIMS *m/z* 357.1187 [M+H]^+^ combined with ^1^H and ^13^C NMR spectroscopic data (Table 1 and Table 2). A comparison of 1D NMR data with those reported for altersolanol O [17] revealed a similar hydroanthraquinone skeleton, while the only differences were the absence of C-1 hydroxy and the replacement of the C-9 carbonyl group with a C-9 hydroxyl group in **5**, which was further confirmed by the key HMBC correlations from H-9 to C-1a and C-9a, and from H_2_-1 to C-1a and C-9 (Figure 2 and Supplementary Materials). The relative configuration was also determined by NOESY correlations and *J* coupling constant analysis. The NOESY correlations from H-9 to H_3_-12 and H_2_-1 indicated that H-9 (ax) and H_3_-12 (ax) were on the same face, showing that the B ring and C ring were *cis* fused with the C-1a and C-4a epoxide ring on the opposite side to H-9 (Figure 3). Further NOESY correlations from H-4 to 2-CH_3_ with the small *J* coupling constant between H-3 and H-4 (^3^*J*_H-3, H-4_ = 3.7 Hz) suggested that H-3 (eq), H-4 (ax), and H_3_-12 (ax) were on the same face of the C ring. The absolute configurations of the stereogenic carbons of **5** were determined as 2*S*, 3*R*, 4*S*, 1a*S*, 4a*S,* 9*S* by a comparison of the experimental and calculated ECD spectra (Figure 6). Compound **5** was named auxarthrol H.

By a comparison of the NMR and MS data with the literature, two known compounds were identified as 4-dehydroxyaltersolanol A (**6**) [14] and altersolanol B (**7**) [11] (Figure 1).

As a typical class of anthraquinone derivatives, auxarthrols characterized with multiple hydroxyl groups attached on the hydroanthraquinone skeleton were first isolated in 1969 [11] and this was followed by total synthesis and biosynthetic studies [18,19]. By the end of 2018, seventeen altersolanols [19] and three auxarthrols [12,19] had been discovered and because of their broad range of biological activities [20,21,22], this class of compounds has received growing attention from the natural product community.

Compounds **1**−**7** were tested for their cytotoxic activity against eleven types of human cancer cell lines using SRB staining [23] and MTT [24] methods, with doxorubicin hydrochloride (Dox) as a positive control. Compounds **1** and **3** showed moderate cytotoxic activity against eleven human cancer cell lines, with IC_50_ values ranging from 4.5 μM to 22.9 μM (Table 3). The antimicrobial activity of **1**–**7** was also evaluated and **1**, **3–4**, and **6−7** showed promising antibacterial activity, with MIC values ranging from 12.5 μM to 200 μM. (Table 4). 

Moreover, all the compounds were investigated for their anticoagulant activity using argatroban as a positive control (inhibition ratio: 65.0%). Compounds **4** and **6** displayed a moderate effect with an inhibition ratio of 47.8% and 51.5%, respectively (Table 5). In addition, all the compounds were tested for antitubercular activity, but only **7** displayed a weak antitubercular effect, with an MIC value of 20.0 μg/mL (Table 6).

## 3. Materials and Methods

### 3.1. General Experimental Procedures

UV spectra were recorded on Waters 2487. IR spectra were recorded on a Nicolet NEXUS 470 spectrophotometer in KBr discs (Thermo Scientific, Beijing, China). Optical rotations were measured on a JASCO P-1020 digital polarimeter (JASCO Corporation, Tokyo, Japan). HRESIMS and ESIMS data were obtained on a Thermo Scientific LTQ Orbitrap XL mass spectrometer. ECD spectra were measured on a JASCO J-715 spectra polarimeter (JASCO Corporation, Tokyo, Japan). NMR spectra were recorded on an Agilent 500 MHz DD2 spectrometer using TMS as the internal standard, and the chemical shifts were recorded as δ values. Semi-preparative HPLC was performed on an ODS column (HPLC (YMC-Pack ODS-A, 10 × 250 mm, 5 μm, 3 mL/min)). MPLC was performed on a Bona-Agela CHEETAHTM HP100 (Beijing Agela Technologies Co., Ltd., Beijing, China). Column chromatography (CC) was performed with silica gel (200–300 mesh, Qingdao Marine Chemical Inc. Qingdao, China) and Sephadex LH-20 (Amersham Biosciences, San Francisco, CA, USA), respectively.

### 3.2. Fungal Material

The fungal strain HDN16-802 was isolated from the sediment sample of Zhangzi Island, collected from Dalian, Liaoning Province, China. The strain was identified as *Sporendonema casei* based on sequencing of the ITS region (GenBank: MK578184). A voucher specimen strain was prepared on potato dextrose agar slants and deposited at −20 °C in the Key Laboratory of Marine Drugs, Chinese Ministry of Education. 

### 3.3. Fermentation and Extraction 

*S. casei* HDN16-802 was cultured on slants with PDA at 28 °C for 7 days. Further fermentation was carried out under static conditions at room temperature for 30 days in Erlenmeyer flasks (1000 mL), with each containing 53 g of oatmeal and naturally collected seawater (125 mL per flask) from Huiquan Bay, Qingdao, China. The pooled fermentation broth, together with mycelium (total of 45 L), were macerated and extracted with an equal volume of EtOAc three times. The organic layers were combined together and concentrated under reduced pressure to yield the extract (10 g). 

### 3.4. Isolation 

The extract (10 g) was fractionated by VLC column chromatography on silica gel using stepwise gradient elution with petroleum ether-CH_2_Cl_2_-MeOH (from PE only to PE with DCM in different ratios and DCM only later, and then from DCM only to DCM with MeOH in different ratios and MeOH only, depending on the polarity from small to large) to give six fractions (fraction 1 to fraction 6). Fraction 5 (eluted with 92:8 DCM-MeOH) was further separated by MPLC and then HPLC, eluting with MeOH/H_2_O (35:65) to obtain **1** (t_R_ 28 min; 10.0 mg). Fraction 2 (eluted with 98:2 DCM-MeOH) was applied on a Sephadex LH-20 column and eluted with MeOH to provide six fractions (fraction 2-1 to fraction 2-6). Fraction 2-4 was separated by HPLC eluting with MeCN/H_2_O (23:77) to obtain **4** (t_R_ 40 min; 5.0 mg) and **7** (t_R_ 35 min; 5.0 mg). Fraction 3 (eluted with 94:6 DCM-MeOH) was further separated by a C-18 ODS column with a step gradient elution of MeOH-H_2_O (15:85-80:20), resulting in four fractions (fraction 3-1 to fraction 3-4). Fraction 3-1 was separated by HPLC eluting with MeCN/H_2_O (gradient 15:85-25:75) to provide **2** (t_R_ 23 min; 2.1 mg), **3** (t_R_ 25 min; 5.1 mg), **5** (t_R_ 45 min; 4.5 mg), and **6** (t_R_ 50 min; 10.1 mg) and Fraction 3-1-3 was further purified by HPLC using MeOH/H_2_O (24:76) as an eluent to obtain **5** (t_R_ 25 min; 7.9 mg).

**Auxarthrol D (1):** Pale yellow crystal; [α]${}_{D}^{25}$ −20.45 (c 0.03, MeOH); UV (MeOH) λ_max_ (log ε) 248 (2.4), 295 (1.2), 350 (1.2) nm; CD (2.5 mM, MeOH) λ_max_ (Δε) 218 (+0.74), 240 (−1.78) nm, 260 (+3.13)nm, 333 (−3.68) nm; IR (KBr) ν_max_ 3366, 2940, 2360, 1700, 1636, 1615, 1385, 1305, 1204, 1164, 1102, 1032, 910 cm^−1^; for ^1^H and ^13^C NMR data, see Table 1 and Table 2; HRESIMS m/z 339.1076 [M+H]^+^ (calculated for C_16_H_19_O_8_, 339.1074)

**Auxarthrol E (2):** Pale yellow powder; [α]${}_{D}^{25}$ −19.0 (c 0.04, MeOH); UV (MeOH) λ_max_ (log ε) 290 (2.4), 330 (1.6) nm; CD (2.5 mM, MeOH) λ_max_ (Δε) 218 (−1.74), 240 (+0.58) nm, 300 (+3.78) nm; IR (KBr) ν_max_ 3356, 2934, 2361, 1717, 1625, 1577, 1376, 1291, 1204, 1158, 1074, 851 cm^−1^; for ^1^H and ^13^C NMR data, see Table 1 and Table 2; HRESIMS m/z 357.1187 [M+H]^+^ (calculated for C_16_H_21_O_9_, 357.1180).

**Auxarthrol F (3):** Pale yellow powder; [α]${}_{D}^{25}$ −65.0 (c 0.2, MeOH); UV (MeOH) λ_max_ (log ε) 280 (2.4), 340 (1.0) nm; CD (2.5 mM, MeOH) λ_max_ (Δε) 218 (−1.54) nm, 240 (+0.78) nm, 300 (+3.12) nm; IR (KBr) ν_max_ 3379, 2925, 2362, 1626, 1375, 1296, 1204, 1160, 1084, 1032, 849, 780 cm^−1^; for ^1^H and ^13^C NMR data, see Table 1 and Table 2; HRESIMS m/z 341.1237 [M+H]^+^ (calculated for C_16_H_21_O_8_, 347.1231).

**Auxarthrol G (4):** Pale yellow powder; [α]${}_{D}^{25}$ +5.38 (c 0.08, MeOH); UV (MeOH) λ_max_ (log ε) 245 (2.4), 300 (1.0), 350 (1.2) nm; CD (2.5 mM, MeOH) λ_max_ (Δε) 210 (+3.74), 267 (−1.78), 333 (2.73) nm; IR(KBr) ν_max_ 3375, 2924, 2359, 1636, 1615, 1385, 1296, 1205, 1162, 1030, 771 cm^−1^; for ^1^H and ^13^C NMR data, see Table 1 and Table 2; HRESIMS m/z 373.0685 [M+H]^+^ (calculated for C_16_H_16_O_8_Cl, 373.0685).

**Auxarthrol H (5):** Pale yellow powder; [α]${}_{D}^{25}$ +12.17 (c 0.2, MeOH); UV (MeOH) λ_max_ (log ε)274 (2.4), 315 (1.4) nm; CD (2.5 mM, MeOH) λ_max_ (Δε) 218 (−1.74), 242 (+0.78) nm, 290 (+2.56) nm, 315 (−1.24) nm, 356 (+0.54) nm; IR (KBr) ν_max_ 3356, 2933, 2361, 1627, 1376, 1298, 1205, 1159, 1103, 1024, 950, 601 cm^−1^; for ^1^H and ^13^C NMR data, see Table 1 and Table 2; HRESIMS m/z 339.1080 [M+H]^+^ (calculated for C_16_H_19_O_8_, 339.1074).

### 3.5. Assay of Cytotoxic Activity

Cytotoxic activity was evaluated as previously reported [25]. 

### 3.6. Assay of Antimicrobial Activity 

Antimicrobial activity was evaluated as previously reported [26].

### 3.7. Assay of Anticoagulant Activity

Anticoagulant activity was evaluated as previously reported [27].

### 3.8. Assay of Antitubercular Activity

Antitubercular activity was evaluated as previously reported [28].

## 4. Conclusions

In conclusion, we reported the isolation and structural elucidation of five new bioactive anthraquinone derivatives (**1**–**5**), together with two known analogues (**6**–**7**), from *Sporendonema casei*. Compound **4** is the second anthraquinone derivative with a chlorine atom. Compounds **1**–**7** were evaluated for their cytotoxic, antimicrobial, anticoagulant, and antitubercular activity. Compounds **1** and **3** showed cytotoxic activities against eleven human cancer cell lines, with IC_50_ values ranging from 4.5 μM to 22.9 μM, while **1**, **3**–**4**, and **6**–**7** showed promising antibacterial activity, with MIC values ranging from 12.5 μM to 200 μM. Compounds **4** and **6** displayed a moderate anticoagulant effect, which are the first anthraquinone derivatives with this activity. In addition, **7** was found to display potential antitubercular activity.

## Figures and Tables

**Table 1:** ^1^H NMR data of compounds **1**–**5** (500 MHz, TMS, *δ* ppm, *J* in Hz).

No.	1 ^a^	2 ^a^	2 ^b^	3 ^a^	4 ^a^	5 ^a^
1	1.86, m	1.92, d (14.6); 1.83, d (14.6)	1.92, d (14.6); 1.83, d (14.6)	1.82, d (14.2); 1.75, d (14.2)	2.24, d (14.8); 1.74, d (14.8)	2.35, dd (15.5); 2.31, dd (15.5)
3	3.61, dd (5.7, 3.1)	3.57, d (3.0)	3.57, m	3.46, t (3.0)	3.61, d (3.6)	3.46, d (3.7)
4	4.22, dd (4.5, 3.1)	4.43, d (3.0)	4.43, t (3.5)	4.39, dd (3.0, 9.7)	4.69, d (3.6)	4.57, d (3.7)
6	6.79, d (2.4)	6.35, d (2.5)	6.35, d (2.5)	6.34, d (2.4)	6.82, d (2.5)	6.42, d (2.4)
8	6.83, d (2.4)	6.64, d (2.5)	6.64, m	6.67, d (2.4)	6.96, d (2.5)	6.67, dd (2.4, 1.2)
9		4.73, s	4.73, d (9.5)	4.52, d (8.7)		4.83, d (1.2)
1a	3.36, (6.0, 1.5)					
4a				2.87, d (9.7)		
11	3.87, s	3.80, s	3.80, s	3.81, s	3.88, s	3.82, s
12	1.14, s	1.20, s	1.20, s	1.19, s	1.27, s	1.21, s
OH-2	4.28, s	5.69, s	5.69, s	5.44, s	6.24, s	
OH-3	4.43, d (5.7)		5.50, d (5.3)	4.81, d (3.0)	4.91, s	
OH-4	5.01, d (4.5)		4.52, d (3.5)	4.57, d (3.0)	4.96, s	
OH-4a	6.46, m	5.56, s	5.57, s			
OH-5	12.44, s	11.97, s	11.97, s	12.37, s	11.15, s	12.19, s
OH-1a		5.29, s	5.29, s	4.91, s	6.97, s	
OH-9			5.30, d (9.5)	5.46, d (8.7)		

^a^ in DMSO; ^b^ in CDCl_3._

**Table 2:** ^13^C NMR data of compounds **1**–**7** (125 MHz, DMSO, TMS, *δ* ppm).

No.	1	2	3	4	5
1	34.2, CH_2_	34.3, CH_2_	38.7, CH_2_	30.7, CH_2_	42.4, CH_2_
2	72.3, C	73.5, C	73.2, C	73.6, C	70.3, C
3	73.7, CH	76.9, CH	75.2, CH	75.7, CH	72.9, CH
4	71.3, CH	64.6, CH	66.1, CH	63.5, CH	65.2, CH
5	166.1, C	164.8, C	163.8, C	163.6, C	164.9, C
6	106.1, CH	99.7, CH	99.5, CH	107.2, CH	100.1, CH
7	166.3, C	166.4, C	166.3, C	165.7, C	166.8, C
8	105.0, CH	106.2, CH	105.8, CH	106.4, CH	106.6, CH
9	197.3, C	70.0, CH	73.9, CH	190.6, C	68.6, CH
10	200.1, C	202.4, C	206.0, C	194.4, C	196.6, C
1a	48.0, CH	79.3, C	78.5, C	80.5, C	64.8, C
4a	78.2, C	78.1, C	53.3, CH	72.1, C	63.3, C
9a	137.6, C	148.4, C	149.0, C	134.5, C	145.9, C
10a	110.1, C	108.6, C	110.8, C	110.0, C	107.1, C
11	56.7, CH_3_	56.1, CH_3_	56.1, CH_3_	56.7, CH_3_	56.2, CH_3_
12	23.1, CH_3_	27.6, CH_3_	27.8, CH_3_	27.6, CH_3_	26.1, CH_3_

**Table 3:** Cytotoxic effect of **1** and **3** against eleven human cancer cell lines.

Comp.	IC_50_ (μM)										
	HL-60	Hela	HCT-116	MGC-803	HO8910	MDA-MB-231	SH-SY5Y	PC-3	BEL-7402	K562	L-02
**1**	7.5	>50.0	14.5	21.8	>50.0	19.1	22.9	21.9	16.6	>50.0	>50.0
**3**	4.5	10.7	7.8	17.7	18.7	10.1	17.2	20.0	21.3	16.5	22.2
Dox ^a^	0.1	0.6	0.2	0.2	0.4	0.2	0.1	1.0	0.4	0.3	0.4

^a^ Dox stands for doxorubicin hydrochloride, which was used as a positive control.

**Table 4:** Antimicrobial effect of **1**–**7** on seven microorganisms.

Comp.	MIC (μM)						
	*Mycobacterium Phlei*	*Proteus Species*	*Bacillus subtilis*	*Candida albicans*	*Vibrio Parahemolyticus*	*Escherichia coli*	*Pseudomonas aeruginosa*
1	25.0	50.0	100	>200	50.0	100	50.0
2	>200	>200	>200	>200	>200	>200	>200
3	200	200	200	>200	>200	>200	200
4	50.0	25.0	25.0	200	100	>200	100
5	>200	>200	>200	>200	>200	>200	>200
6	25.0	50.0	25.0	>200	25.0	>200	25.0
7	25.0	100	25.0	>200	25.0	>200	12.5
Positive Control	3.12 ^a^	1.56 ^a^	0.781 ^a^	1.56 ^b^	0.781 ^a^	0.391 ^a^	1.56 ^a^

^a^ Ciprofloxacin used as a positive control for bacteria; ^b^ Nystatin used as a positive control for *Candida albicans*.

**Table 5:** Anticoagulant activity of **1**–**7**.

Comp.	1	2	3	4	5	6	7	Argatroban ^b^
Inhibition ratio ^a^	12.5	19.9	14.4	47.8	27.3	51.5	19.3	65.0

^a^ Data are expressed as inhibition ratio values (%); ^b^ Argatroban was used as a positive control.

**Table 6:** Antitubercular activity of **1**–**7** against AlRa.

Comp.	1	2	3	4	5	6	7	Rifampin ^b^
MIC ^a^	>20.0	>20.0	>20.0	>20.0	>20.0	>20.0	20.0	1.0

^a^ Data are expressed as MIC values (μg/mL); ^b^ Rifampin was used as a positive control.

## Supplementary Materials

The following are available online at https://www.mdpi.com/1660-3397/17/6/334/s1, 1D and 2D NMR and HRESIMS spectra of **1**–**7**. Figures S1–S8: 1D and 2D NMR and HRESIMS spectra of Auxarthrol D (**1**); Figures S9–S17: 1D and 2D NMR and HRESIMS spectra of Auxarthrol E (**2**); Figures S18–S25: 1D and 2D NMR and HRESIMS spectra of Auxarthrol F (**3**); Figures S26–S33: 1D and 2D NMR and HRESIMS spectra of Auxarthrol G (**4**); Figures S34–S41: 1D and 2D NMR and HRESIMS spectra of Auxarthrol H (**5**).
