# Supplementary material for: Anthraquinone Derivatives from a Marine-Derived Fungus Sporendonema casei HDN16-802
Source: Mar Drugs. 2019 Jun 4;17(6):334. doi: 10.3390/md17060334 (PMC6627905; doi:10.3390/md17060334)

Anthraquinone Derivatives from a Marine-Derived Fungus *Sporendonema casei* HDN16-802

Xueping Ge [1], Chunxiao Sun [1], Yanyan Feng [1], Lingzhi Wang [1], Jixing Peng [3], Qian Che [1], Qianqun Gu [1], Tianjiao, Zhu [1, 2], Dehai Li [1,2] and Guojian Zhang [1,2,*]

[1] Key Laboratory of Marine Drugs, Chinese Ministry of Education, School of Medicine and Pharmacy, Ocean University of China, Qingdao 266003, China; 15610568273@163.com (X.G.); sunchunxiao93@163.com (C.S.); yy15715321143@163.com (Y.F.); 952172659@qq.com (L.W.); pengjixing1987@163.com (J.P.); cheqian064@ouc.edu.cn (Q.C.); guqianq@ouc.edu.cn (Q.G.) zhutj@ouc.edu.cn (T.Z.); dehaili@ouc.edu.cn (D.L.)

[2] Laboratory for Marine Drugs and Bioproducts of Qingdao National Laboratory for Marine Science and Technology, Qingdao, 266237, China

[3] Key Laboratory of Testing and Evaluation for Aquatic Product Safety and Quality, Ministry of Agriculture and Rural Affairs, Yellow Sea Fisheries Research Institute, Chinese Academy of Fishery Sciences, Qingdao 266071, China.

[*] Correspondence: zhangguojian@ouc.edu.cn; Tel.: +86 532-82032065.

List of Supporting Information

Figure S1. ^{1}H NMR spectrum (500 MHz) of auxarthrol D (**1**) in DMSO-d_6.............S3
Figure S2. ^{13}C NMR spectrum (125 MHz) of auxarthrol D (**1**) in DMSO-d_6............S3
Figure S3. DEPT spectrum (125 MHz) of auxarthrol D (**1**) in DMSO-d_6.....................S4
Figure S4. ^{1}H-^{1}H COSY spectrum of auxarthrol D (**1**) S4
Figure S5. HSQC spectrum of auxarthrol D (**1**) S5
Figure S6. HMBC spectrum of auxarthrol D (**1**) S5
Figure S7. NOE spectrum of auxarthrol D (**1**) S6
Figure S8. HRESIMS spectrum of auxarthrol D (**1**) S7
Figure S9. ^{1}H NMR spectrum (500 MHz) of auxarthrol E (**2**) in DMSO-d_6................S7
Figure S10. ^{1}H NMR spectrum (500 MHz) of auxarthrol E (**2**) in $CDCl_3$. S8
Figure S11. ^{13}C NMR spectrum (125 MHz) of auxarthrol E (**2**) in DMSO-d_6............S8
Figure S12. DEPT spectrum (125 MHz) of auxarthrol E (**2**) in DMSO-d_6..................S9
Figure S13. ^{1}H-^{1}H COSY spectrum of auxarthrol E (**2**) S9
Figure S14. HSQC spectrum of auxarthrol E (**2**) S10
Figure S15. HMBC spectrum of auxarthrol E (**2**) S10
Figure S16. NOE spectrum of auxarthrol E (**2**) S11
Figure S17. HRESIMS spectrum of auxarthrol E (**2**) S12
Figure S18. ^{1}H NMR spectrum (500 MHz) of auxarthrol F (**3**) in DMSO-d_6...........S13
Figure S19. ^{13}C NMR spectrum (125 MHz) of auxarthrol F (**3**) in DMSO-d_6..........S13
Figure S20. DEPT spectrum (125 MHz) of auxarthrol F (**3**) in DMSO-d_6................S14
Figure S21. ^{1}H-^{1}H COSY spectrum of auxarthrol F (**3**) S14
Figure S22. HSQC spectrum of auxarthrol F (**3**) S15
Figure S23. HMBC spectrum of auxarthrol F (**3**) S15
Figure S24. NOE spectrum of auxarthrol F (**3**) S16
Figure S25. HRESIMS spectrum of auxarthrol F (**3**) S17
Figure S26. ^{1}H NMR spectrum (500 MHz) of auxarthrol G (**4**) in DMSO-d_6..........S18
Figure S27. ^{13}C NMR spectrum (125 MHz) of auxarthrol G (**4**) in DMSO-d_6..........S18
Figure S28. DEPT spectrum (125 MHz) of auxarthrol G (**4**) in DMSO-d_6................S19
Figure S29. ^{1}H-^{1}H COSY spectrum of auxarthrol G (**4**) S19
Figure S30. HSQC spectrum of auxarthrol G (**4**) S20
Figure S31. HMBC spectrum of auxarthrol G (**4**) S20
Figure S32. NOE spectrum of auxarthrol G (**4**) S21
Figure S33. HRESIMS spectrum of auxarthrol G (**4**) S22
Figure S34. ^{1}H NMR spectrum (500 MHz) of auxarthrol H (**5**) in DMSO-d_6...........S22
Figure S35. ^{13}C NMR spectrum (125 MHz) of auxarthrol H (**5**) in DMSO-d_6..........S23
Figure S36. DEPT spectrum (125 MHz) of auxarthrol H (**5**) in DMSO-d_6................S23
Figure S37. ^{1}H-^{1}H COSY spectrum of auxarthrol H (**5**) S24
Figure S38. HSQC spectrum of auxarthrol H (**5**) S24
Figure S39. HMBC spectrum of auxarthrol H (**5**) S25
Figure S40. NOE spectrum of auxarthrol H (**5**) S25
Figure S41. HRESIMS spectrum of auxarthrol H (**5**) S26

Figure S1. ^{1}H NMR spectrum (500 MHz) of auxarthrol D (**1**) in DMSO-d_6.

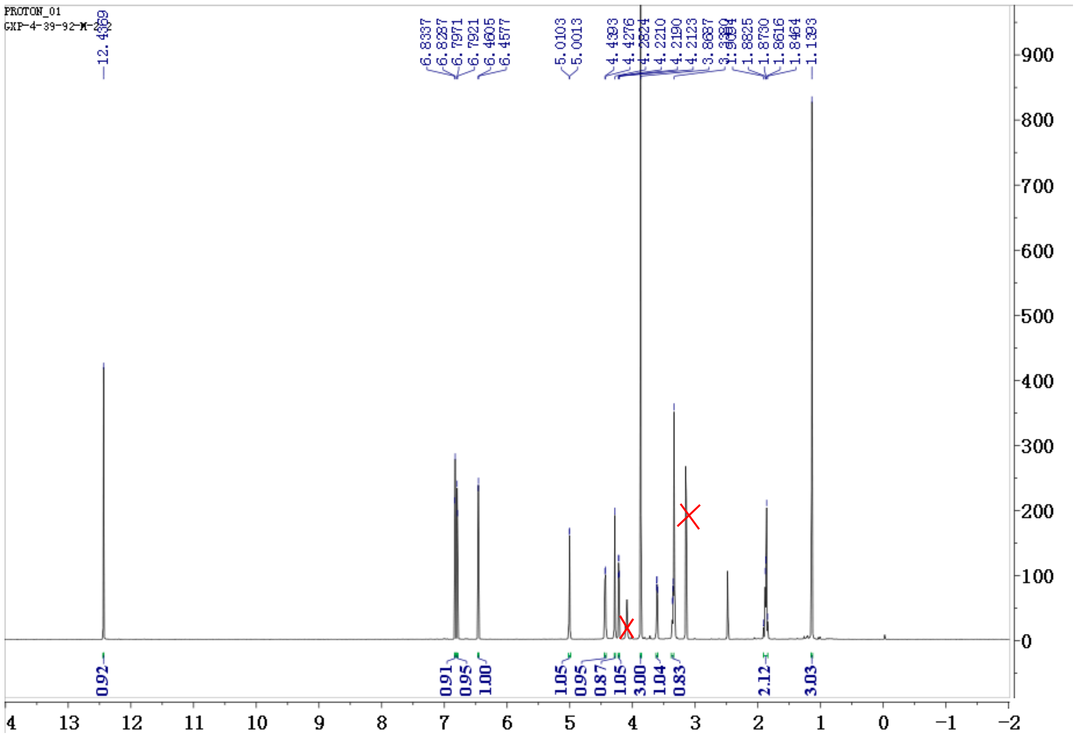

Figure S2. ^{13}C NMR spectrum (125 MHz) of auxarthrol D (**1**) in DMSO-d_6.

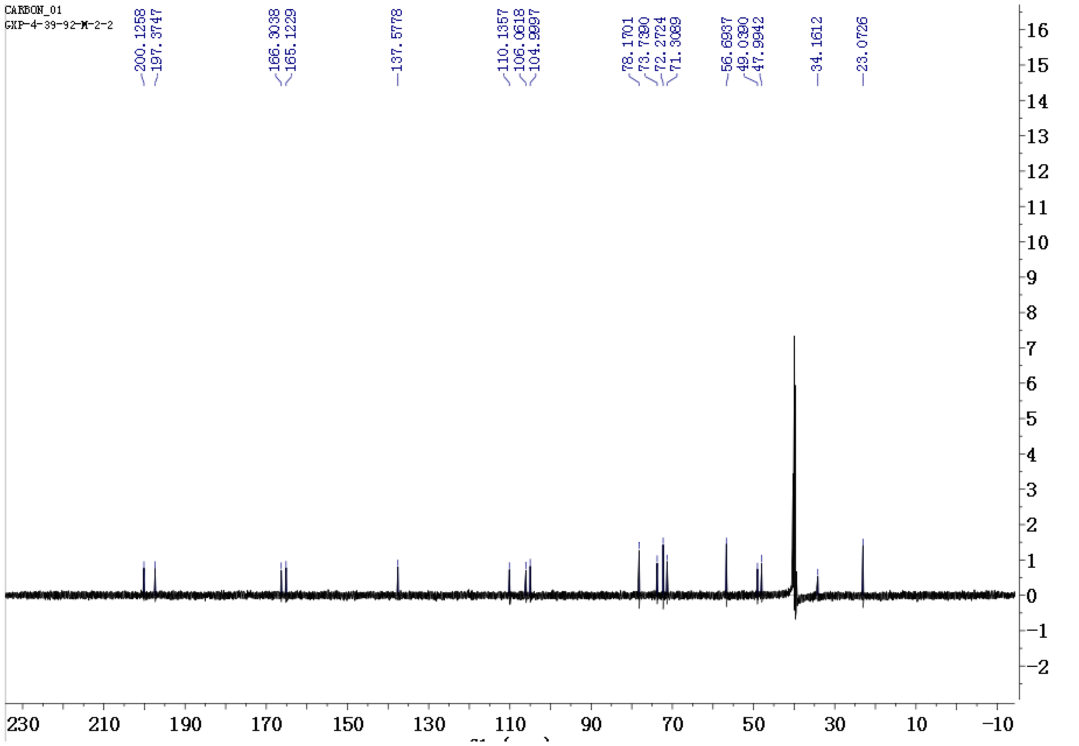

Figure S3. DEPT spectrum (125 MHz) of auxarthrol D (**1**).

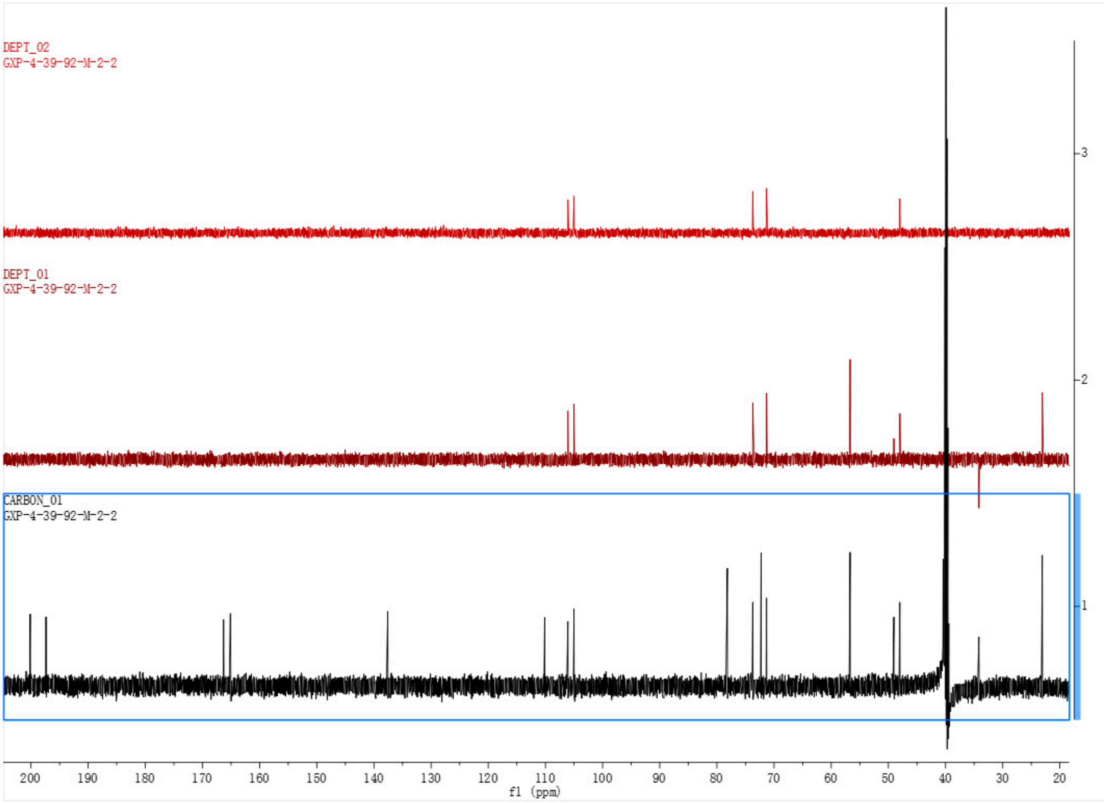

Figure S4. H-H COSY spectrum of auxarthrol D (**1**).

.

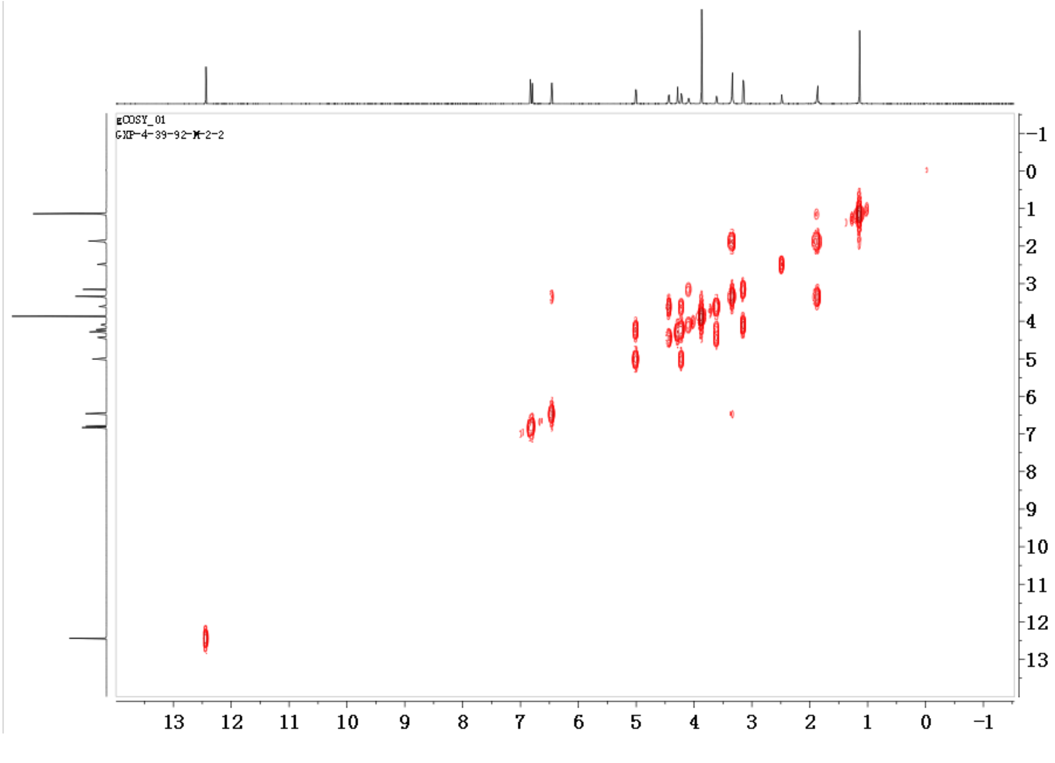

Figure S5. HSQC spectrum of auxarthrol D (**1**).

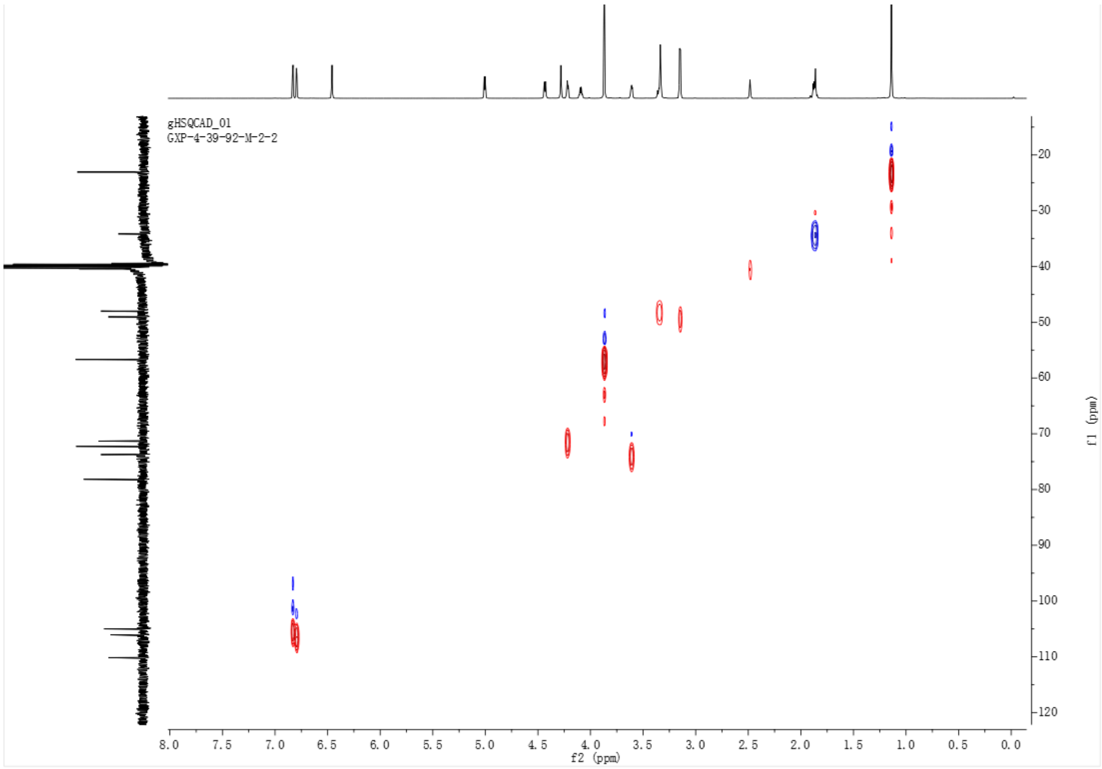

Figure S6. HMBC spectrum of auxarthrol D (**1**).

.

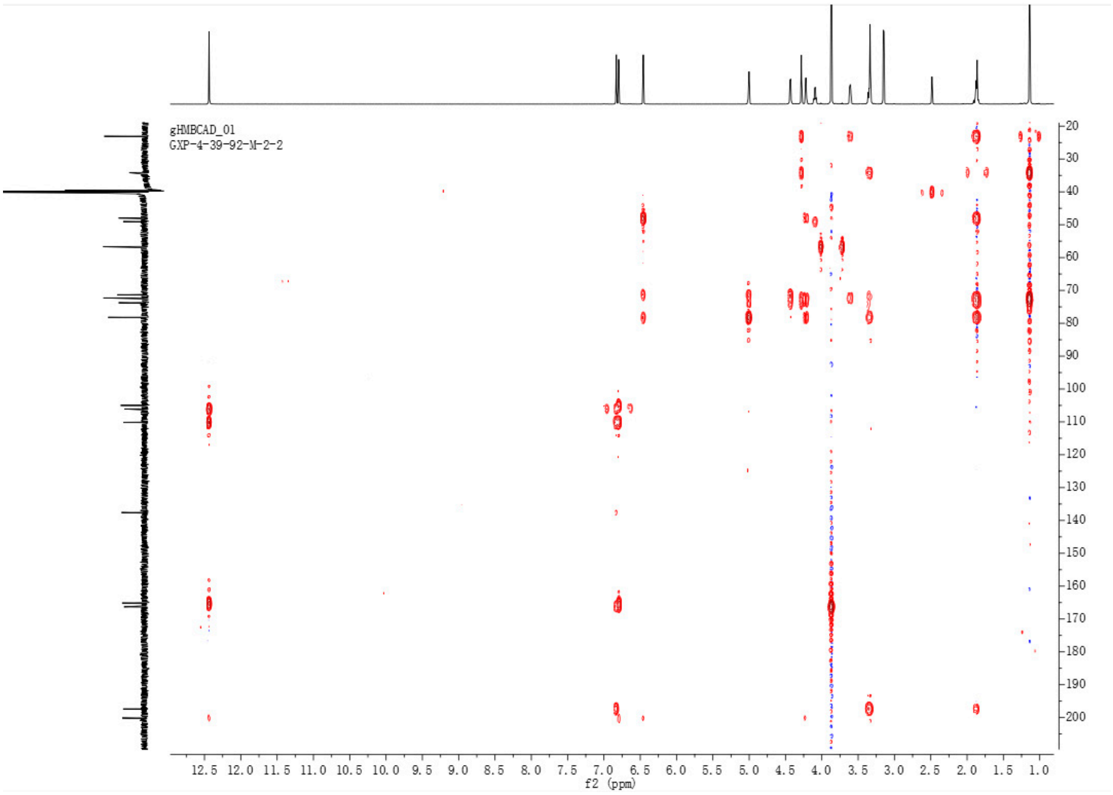

Figure S7. NOE spectrum of auxarthrol D (**1**).

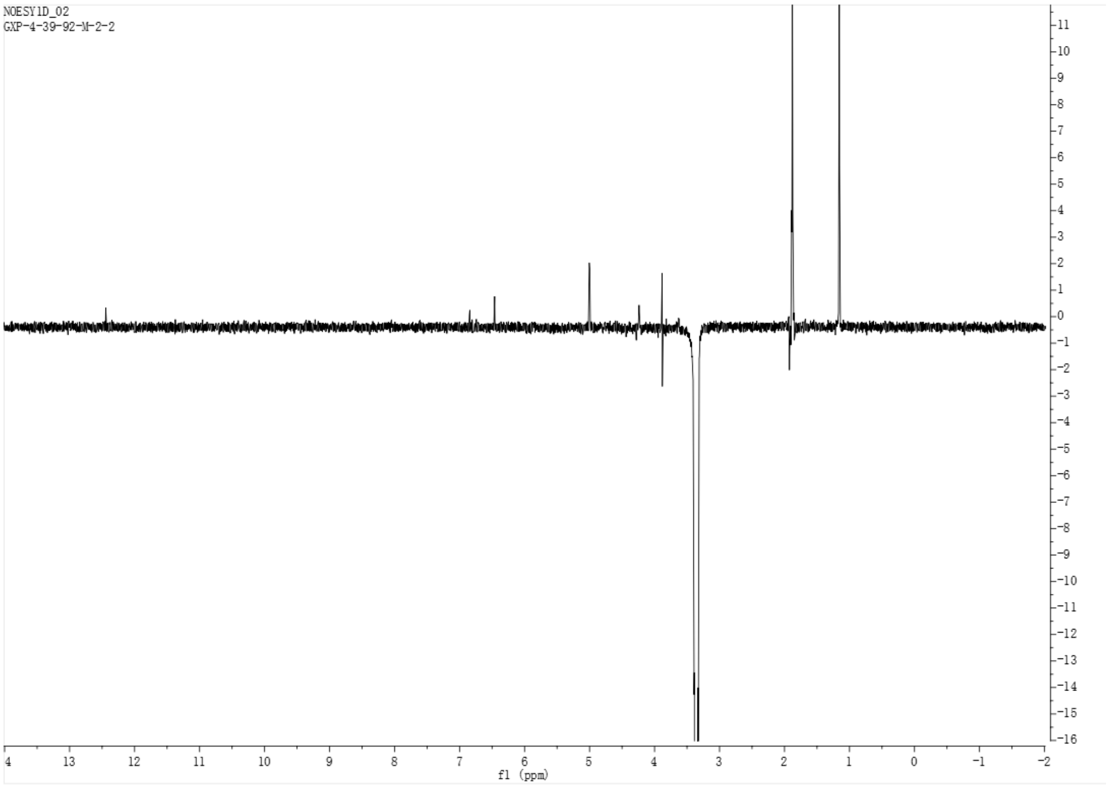

.

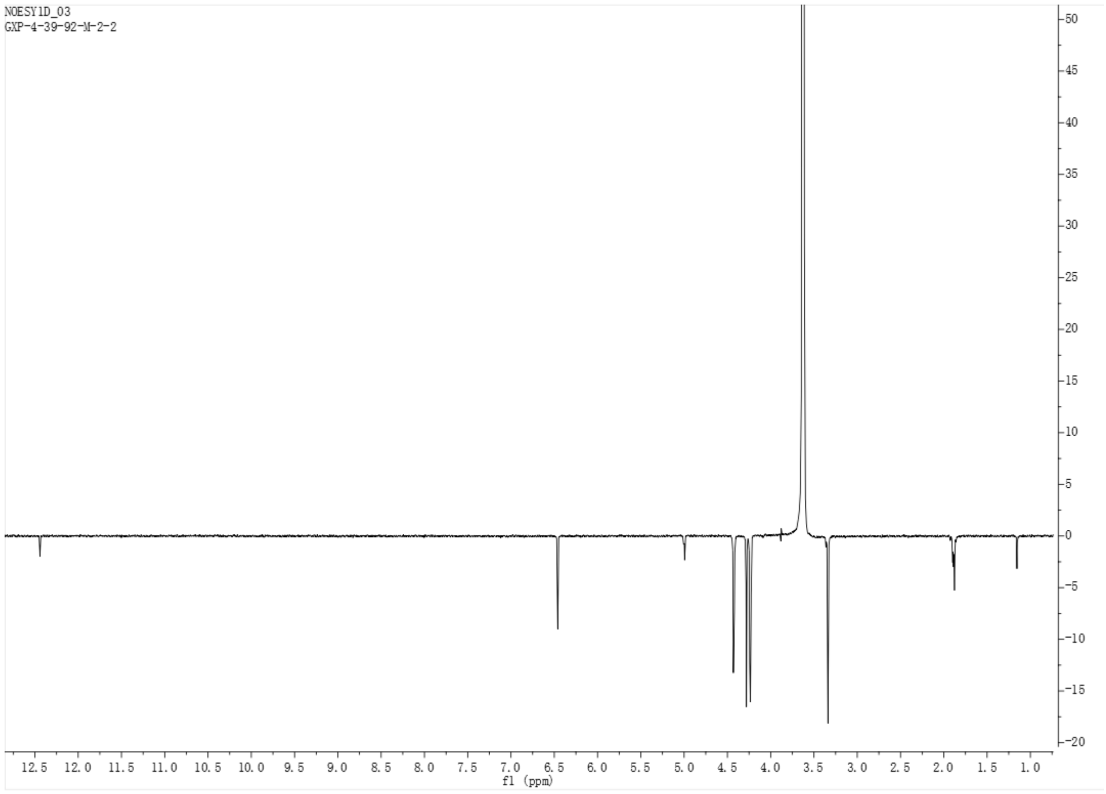

Figure S8. HRESIMS of auxarthrol D (**1**).

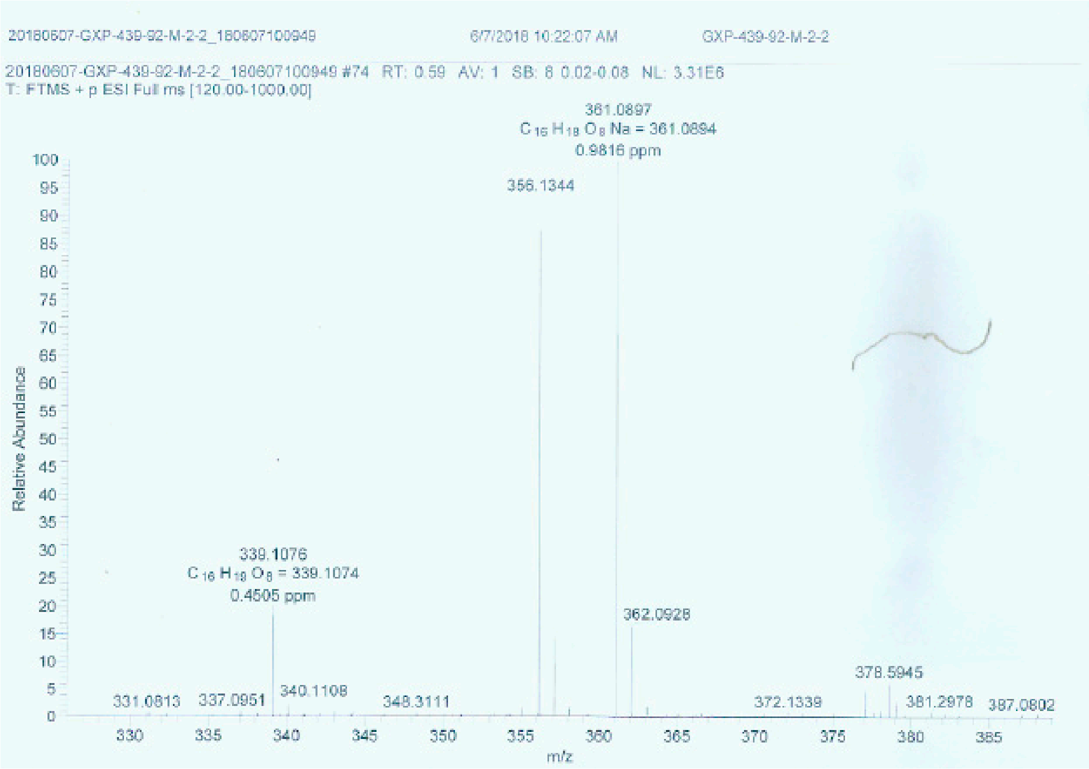

Figure S9. ^{1}H NMR spectrum (500 MHz) of auxarthrol E (**2**) in DMSO-d_6.

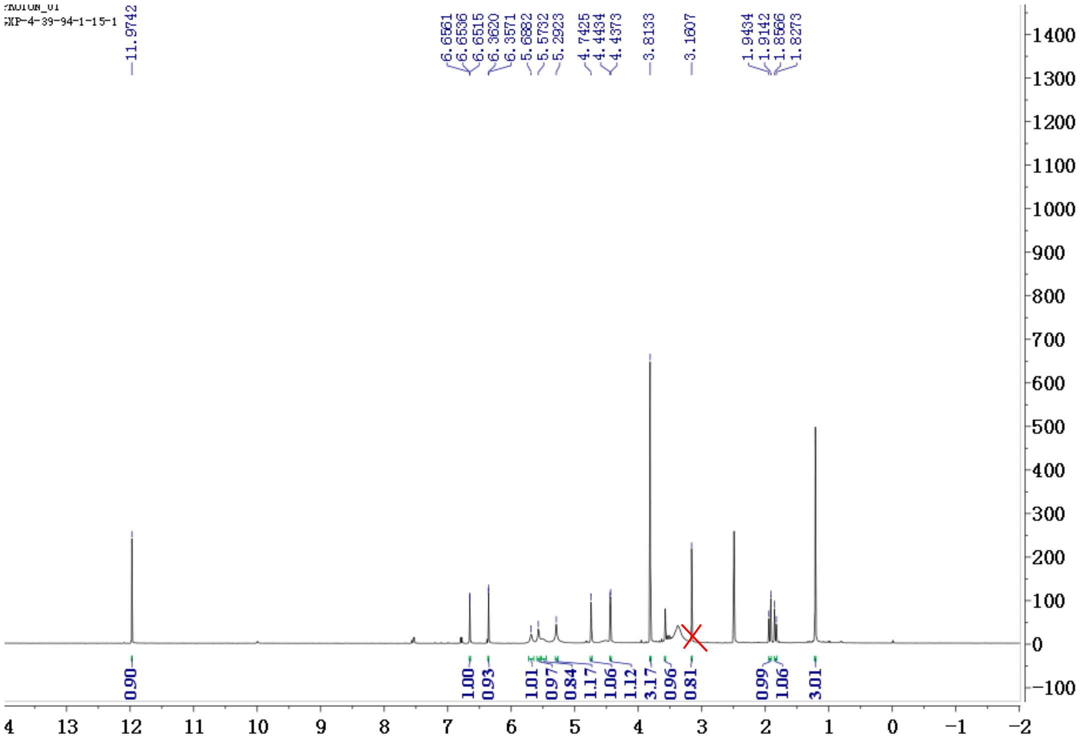

Figure S10. ^{1}H NMR spectrum (500 MHz) of auxarthrol E (**2**) in $CDCl_3$.

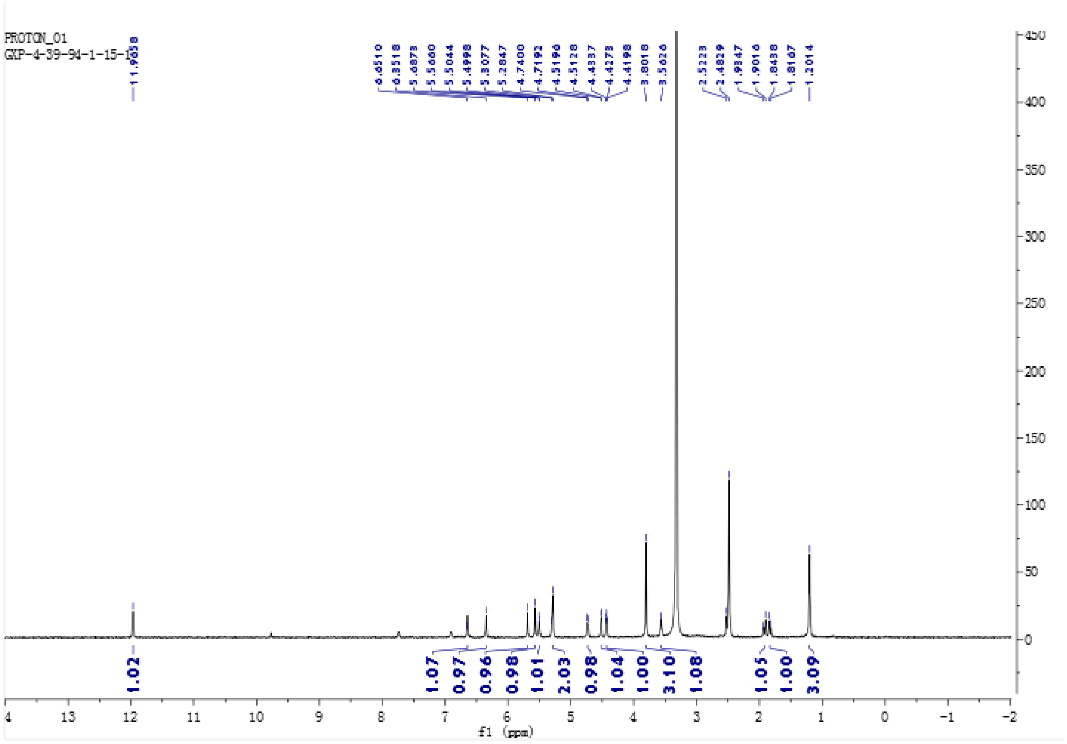

Figure S11. ^{13}C NMR spectrum (125 MHz) of auxarthrol E (**2**).

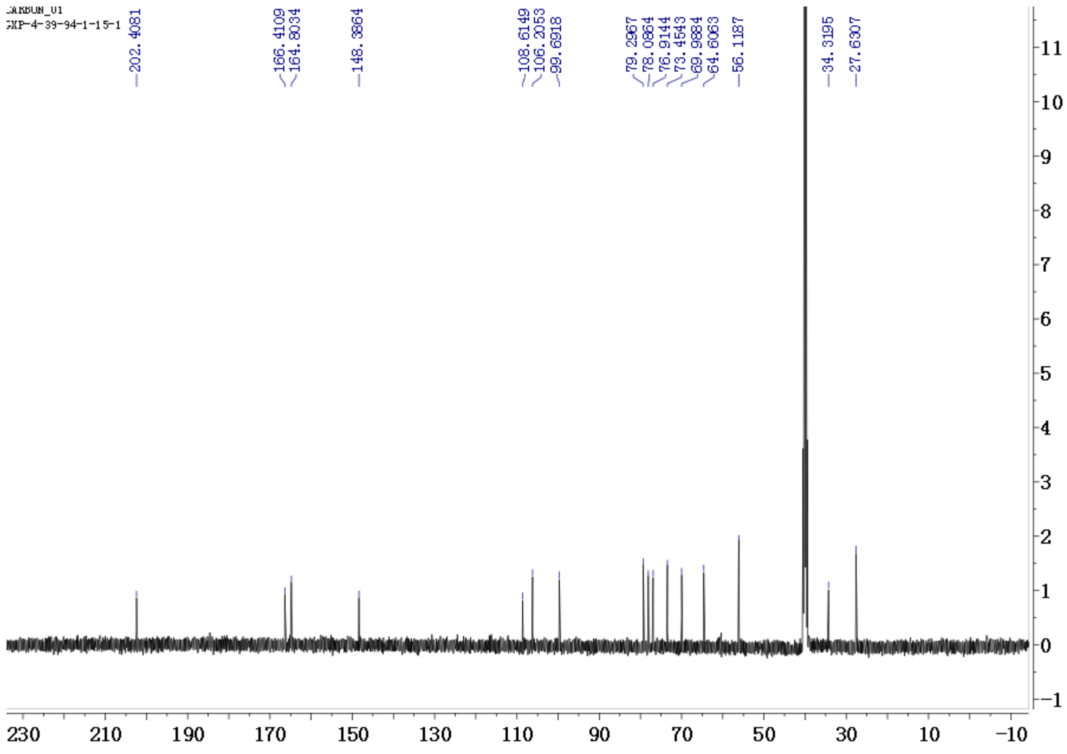

Figure S12. DEPT spectrum (125 MHz) of auxarthrol E (**2**).

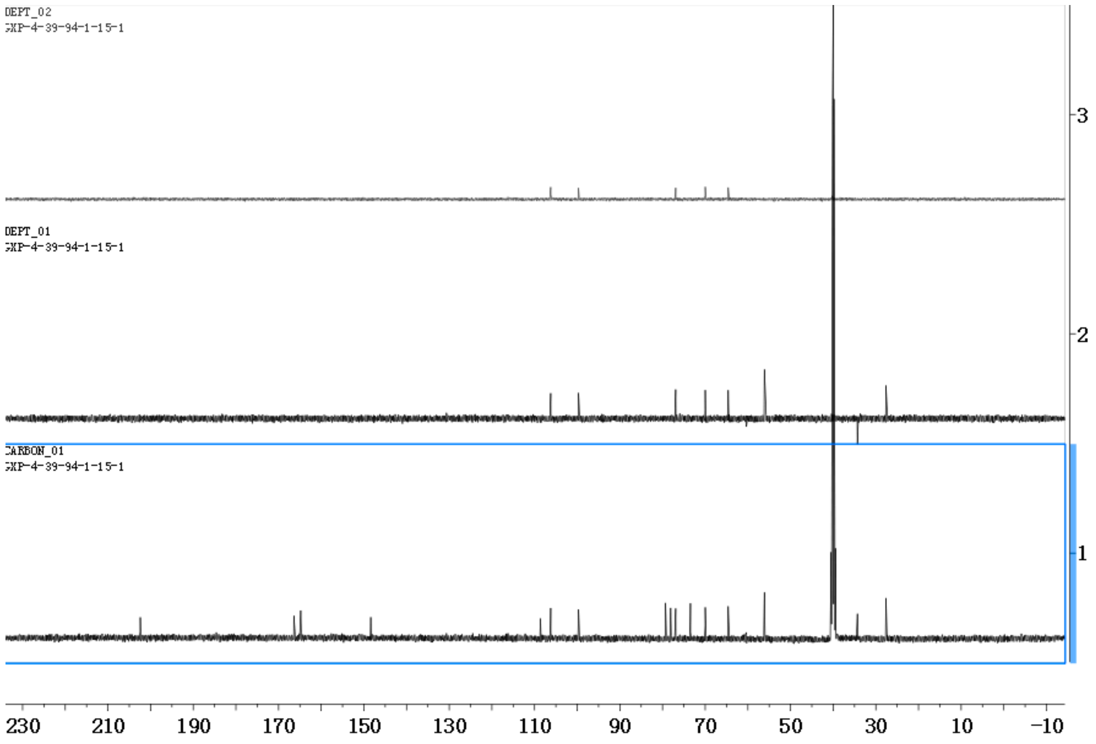

Figure S13. H-H COSY spectrum of auxarthrol E (**2**).

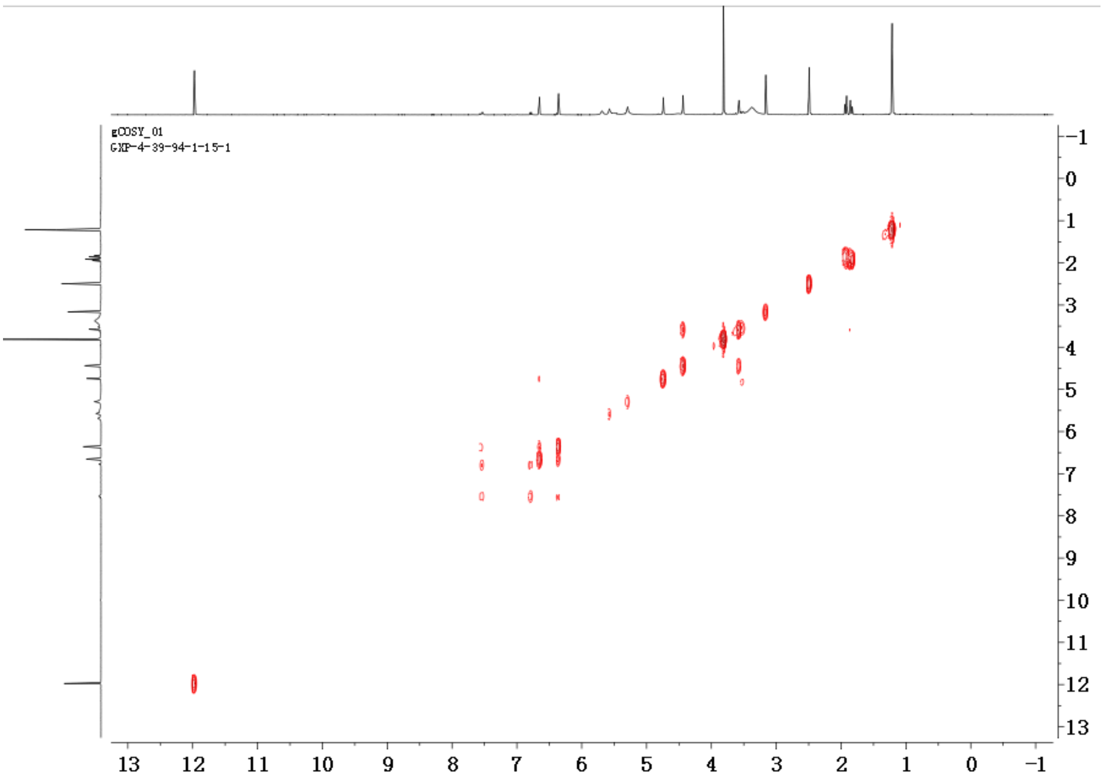

Figure S14. HSQC spectrum of auxarthrol E (**2**).

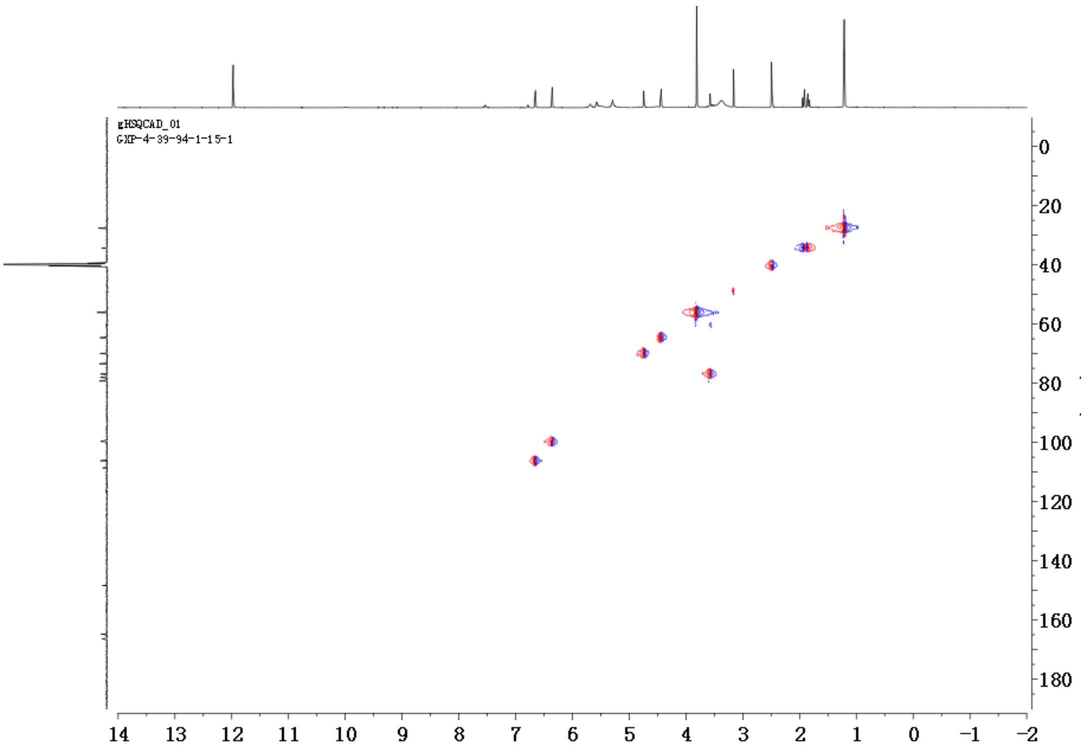

Figure S15. HMBC spectrum of auxarthrol E (**2**).

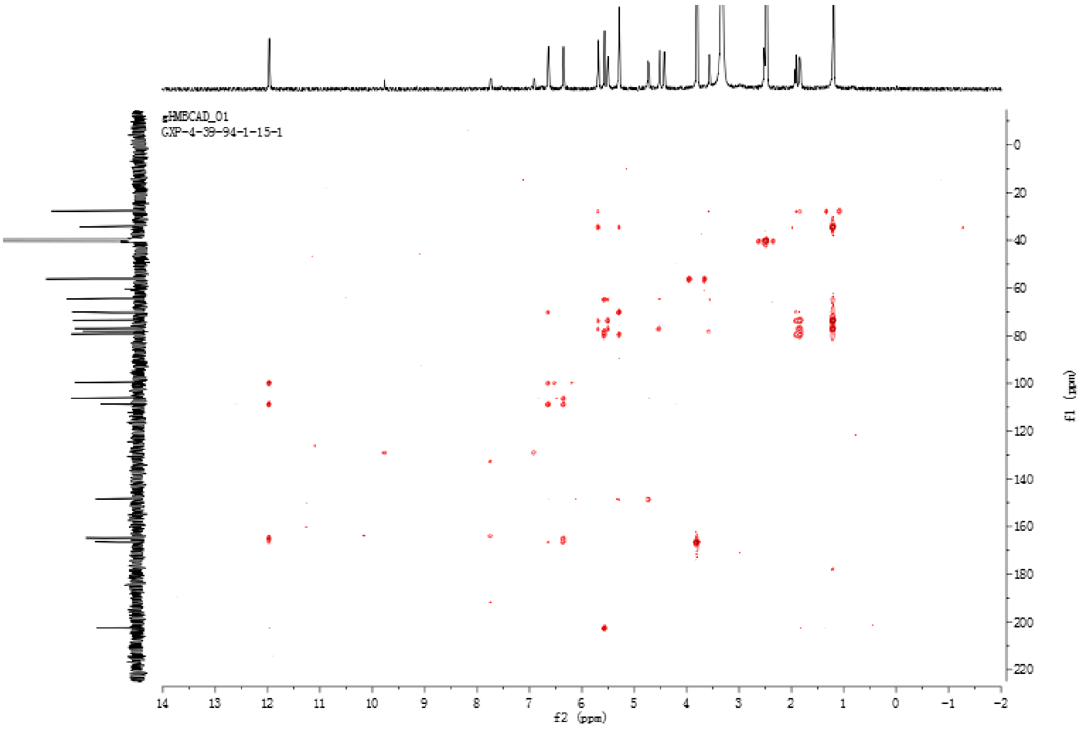

Figure S16. NOE spectrum of auxarthrol E (**2**).

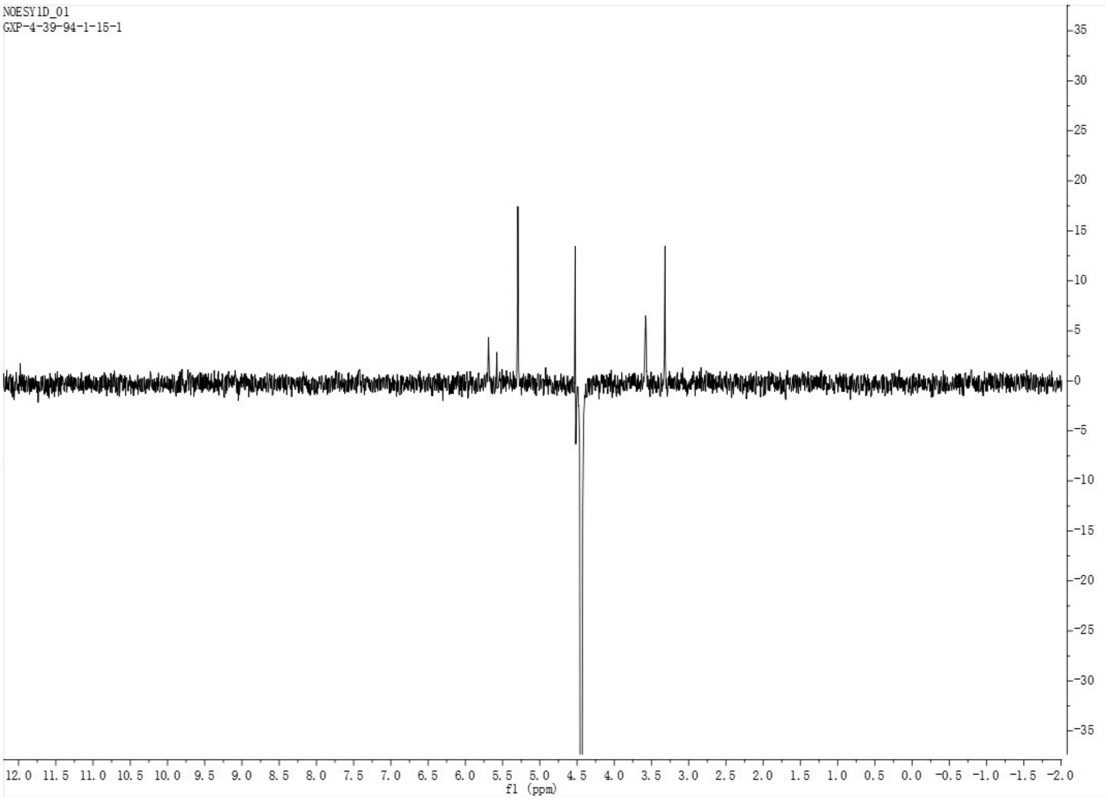

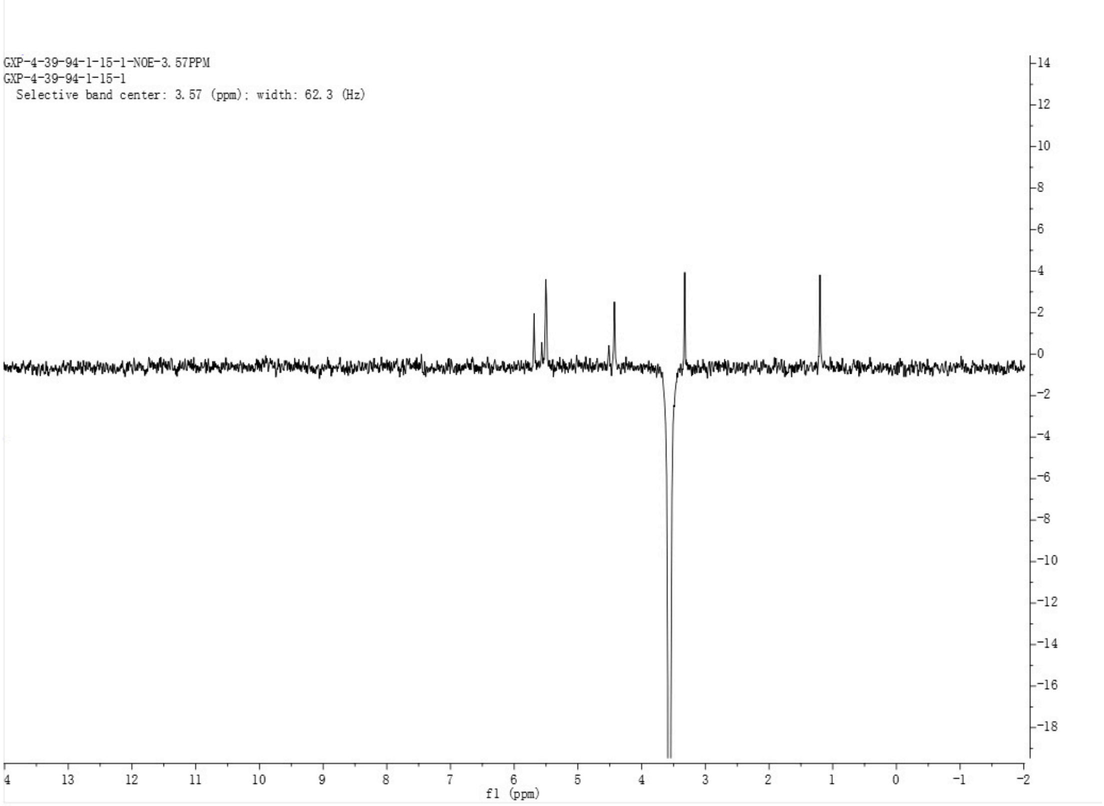

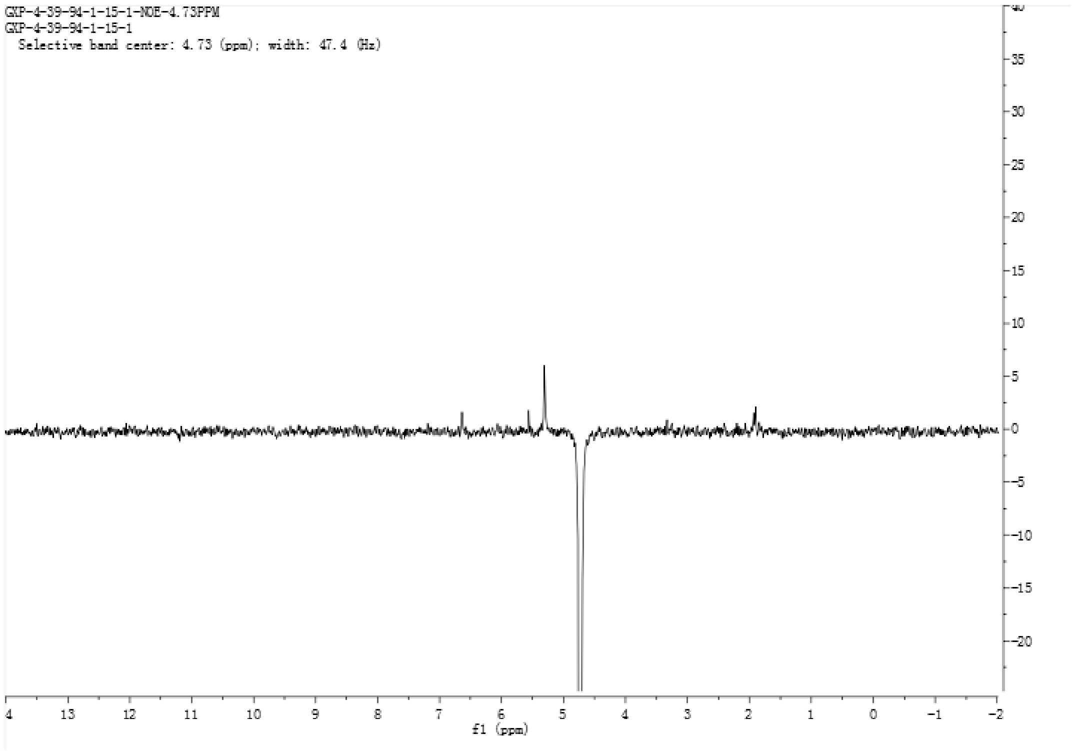

Figure S17. HRESIMS of auxarthrol E (**2**).

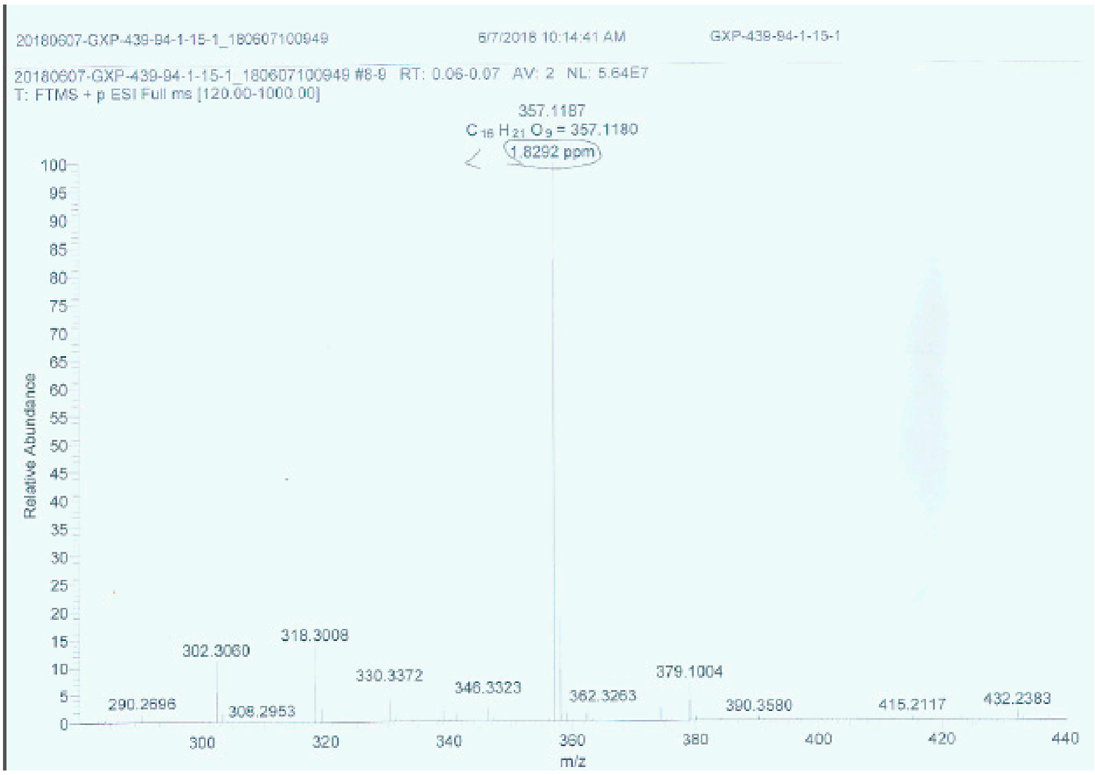

Figure S18. ^{1}H NMR spectrum (500 MHz) of auxarthrol F (**3**) in DMSO-d_6.

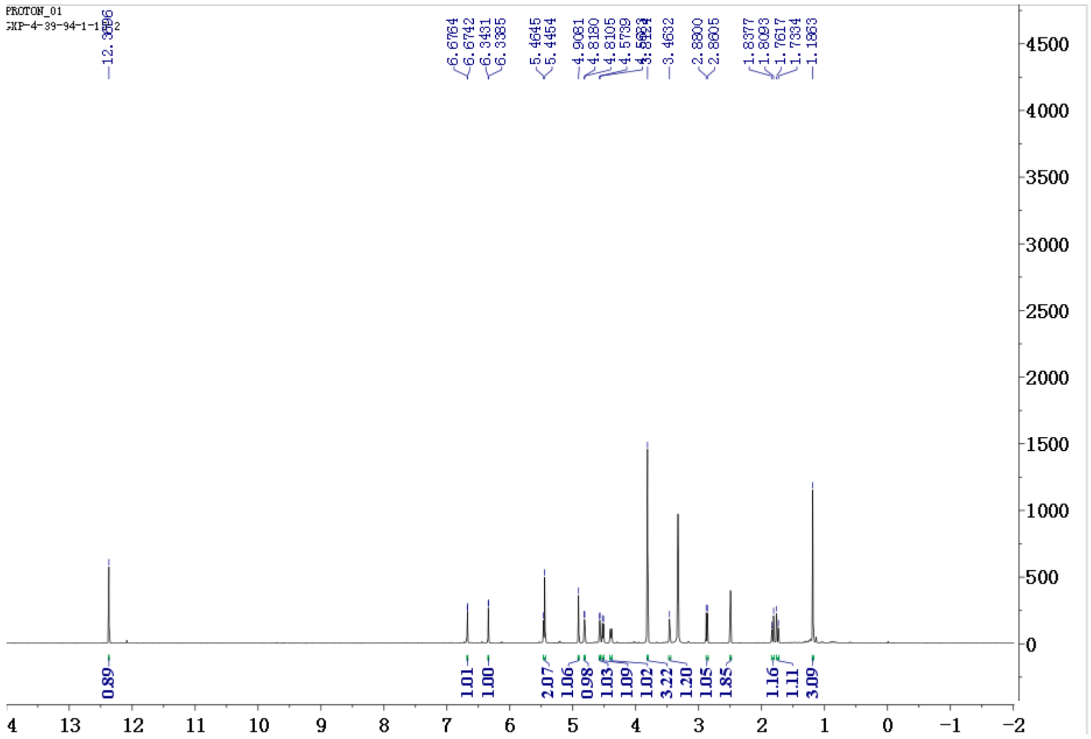

Figure S19. ^{13}C NMR spectrum (125 MHz) of auxarthrol F (**3**) in DMSO-d_6.

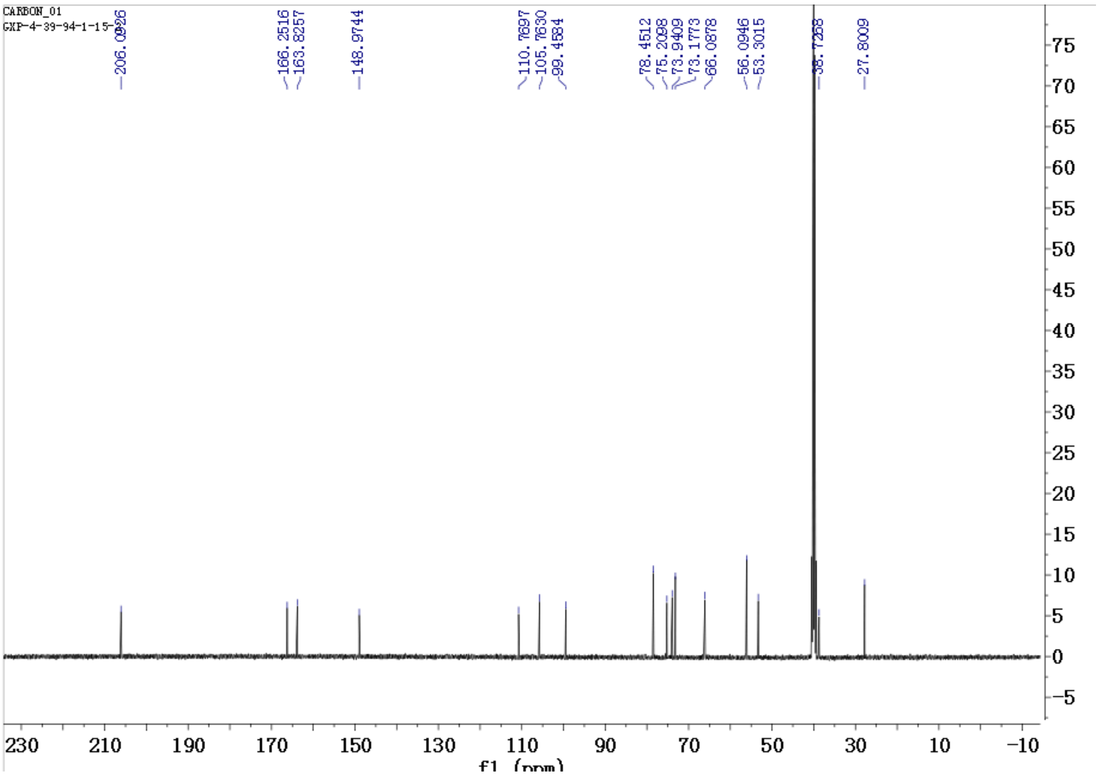

Figure S20. DEPT (125 MHz) spectrum of auxarthrol F (**3**).

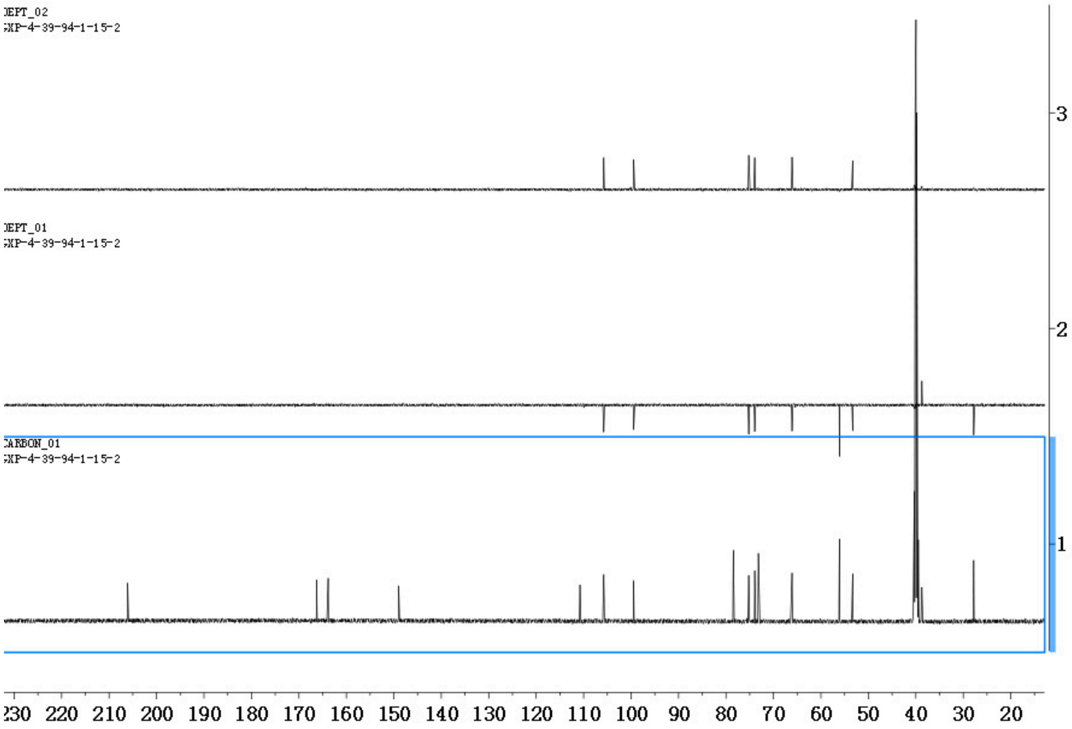

Figure S21. H-H COSY spectrum of auxarthrol F (**3**).

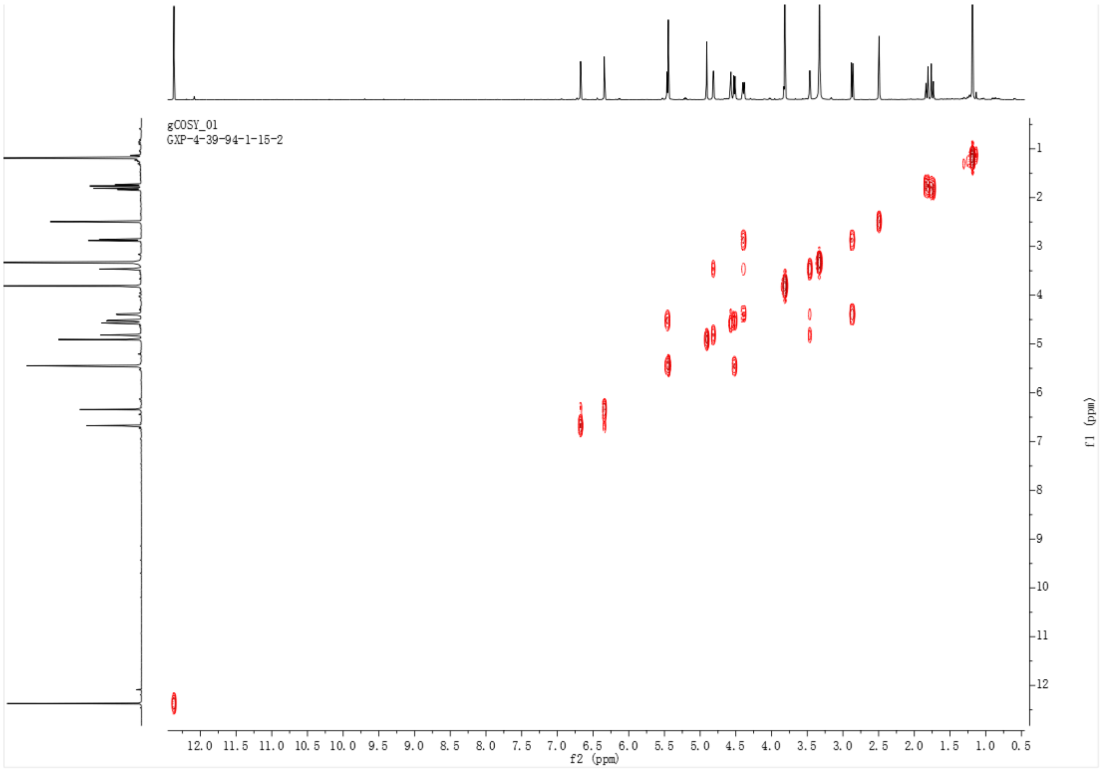

Figure S22. HSQC spectrum of auxarthrol F (**3**).

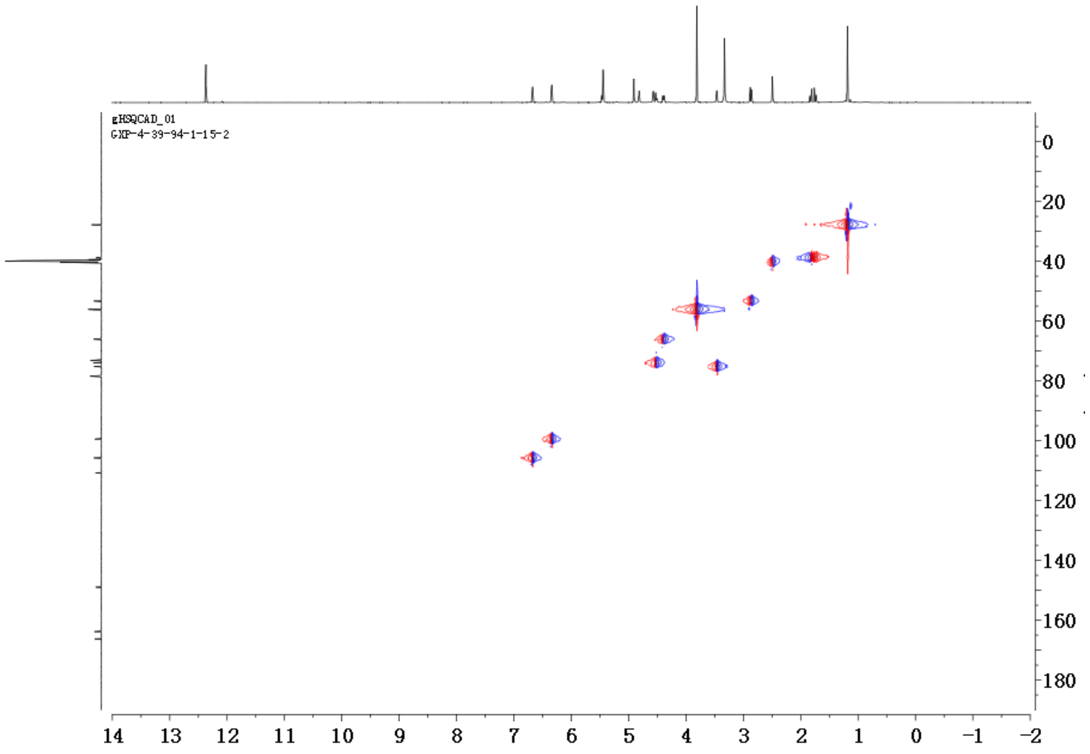

Figure S23. HMBC spectrum of auxarthrol F (**3**).

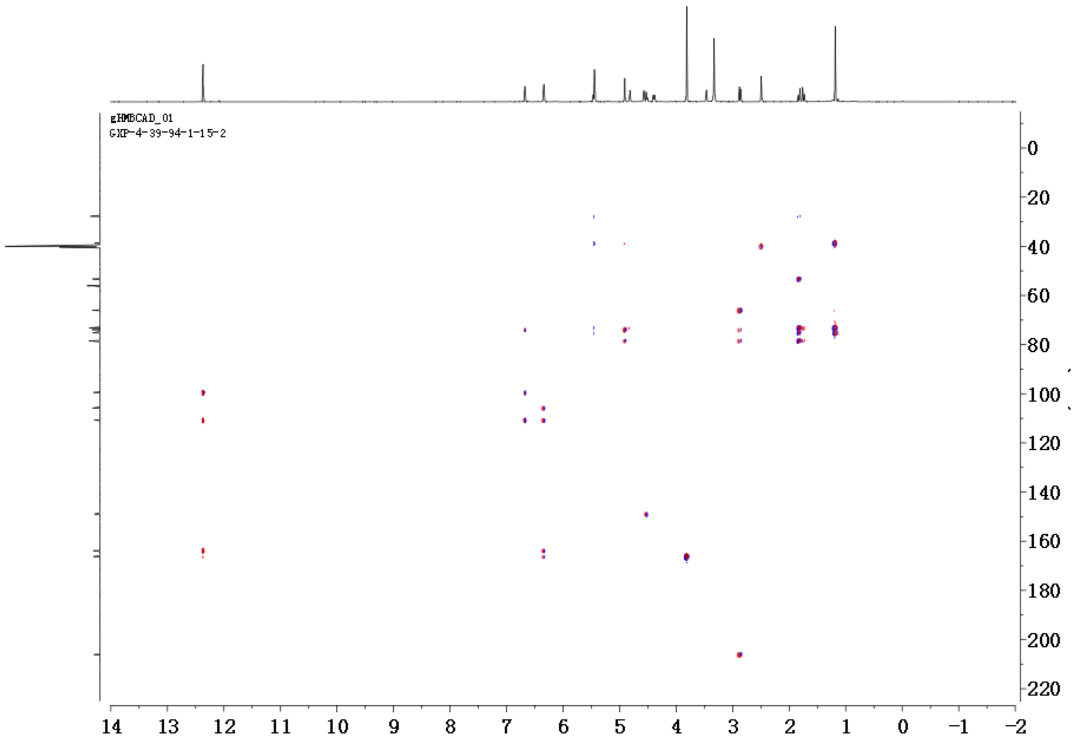

Figure S24. NOE spectrum of auxarthrol F (**3**).

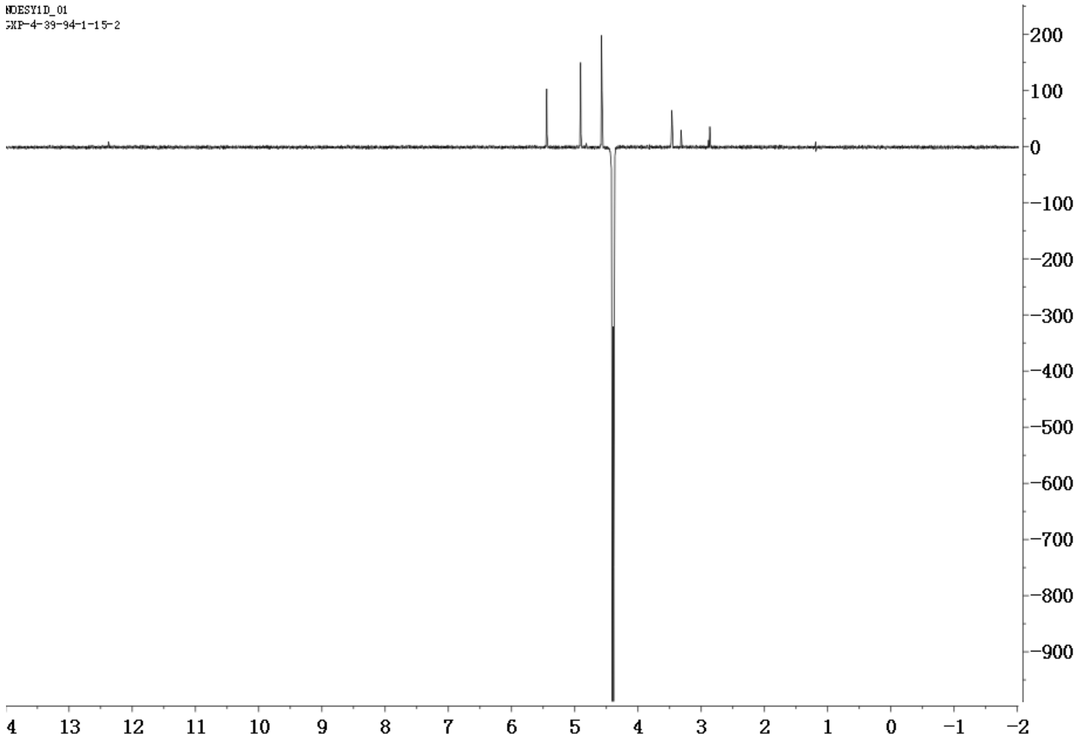

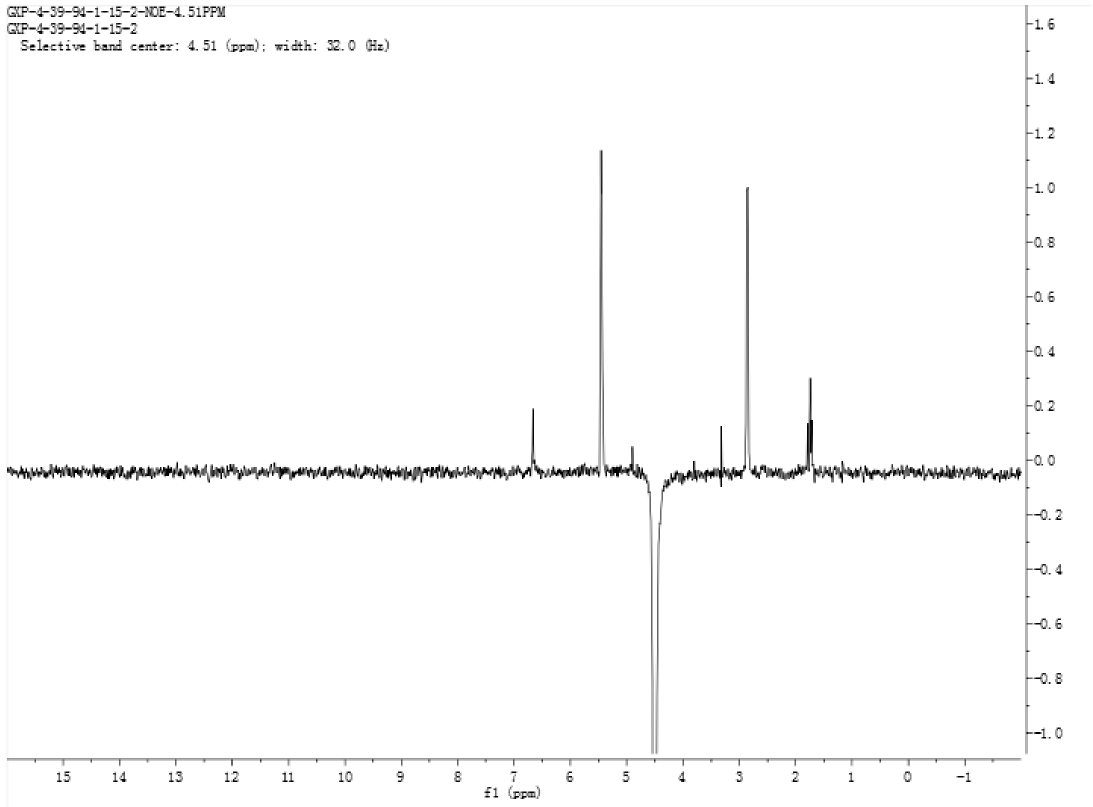

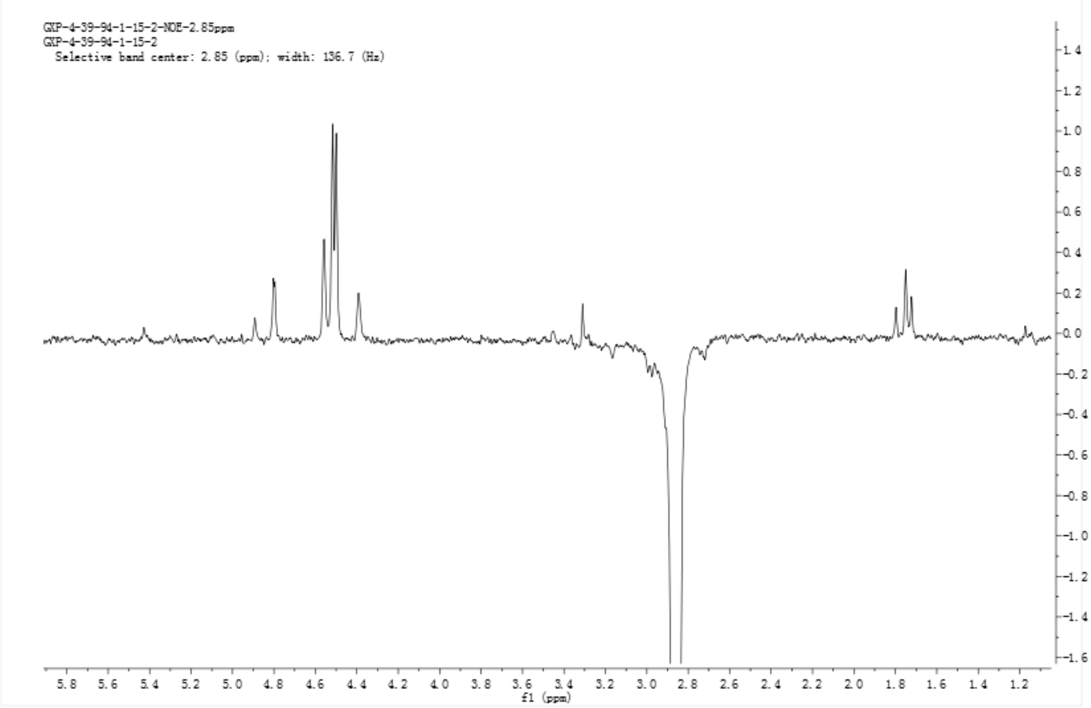

Figure S25. HRESIMS of auxarthrol F (**3**).

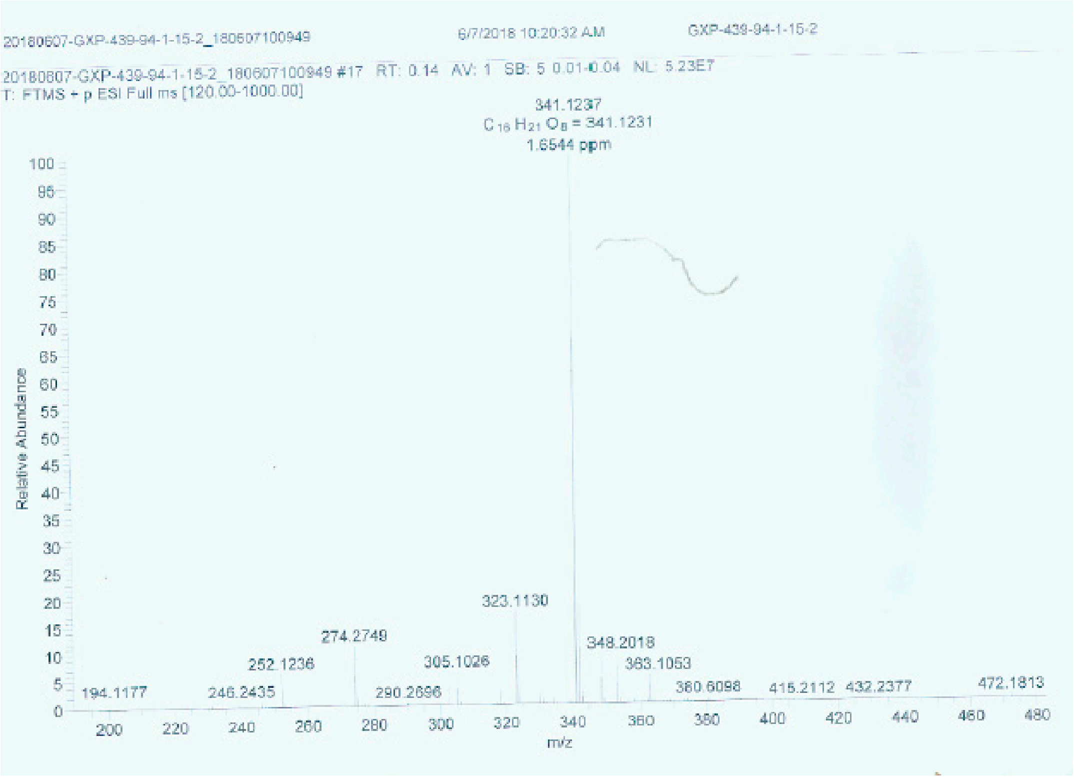

Figure S26. ^{1}H NMR spectrum (500 MHz) of auxarthrol G (**4**) in DMSO-*d*$_6$.

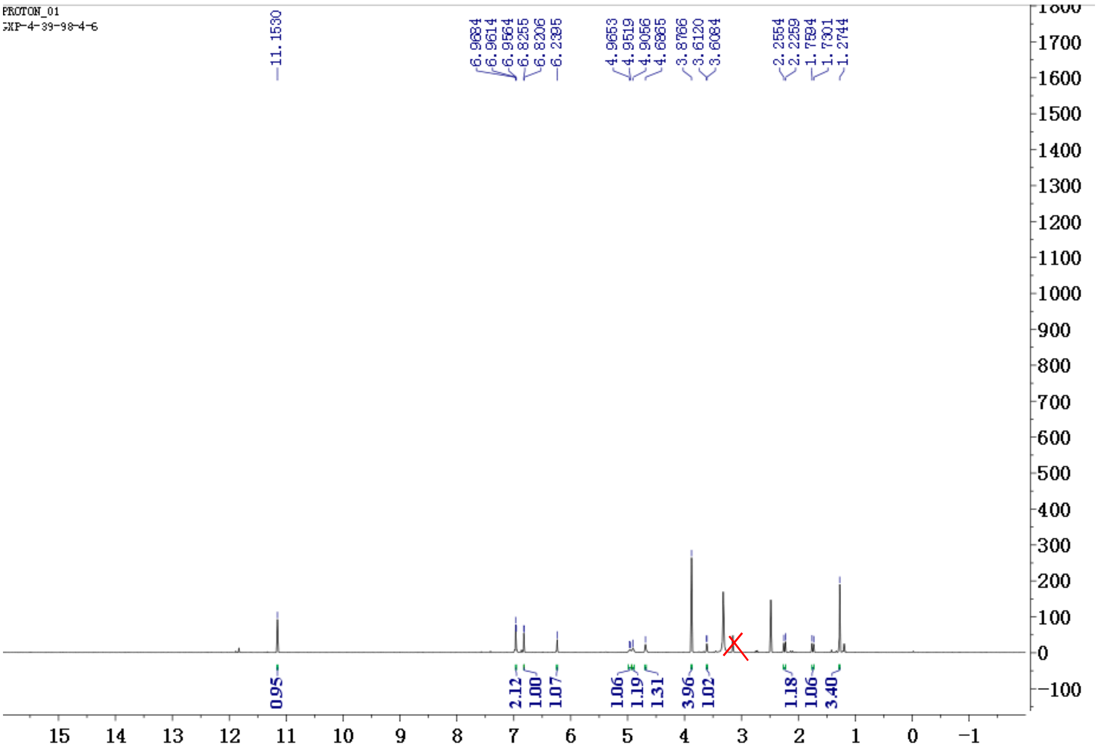

Figure S27. ^{13}C NMR spectrum (125 MHz) of auxarthrol G (**4**) in DMSO-*d*$_6$.

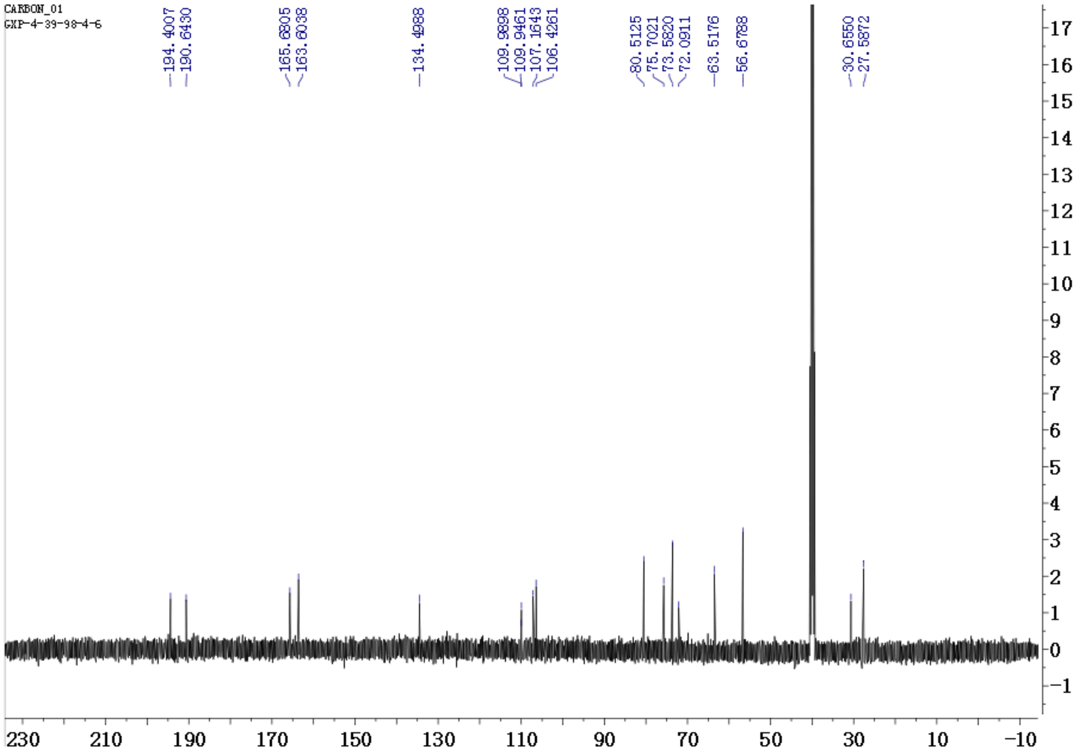

Figure S28. DEPT (125 MHz) spectrum of auxarthrol G (**4**).

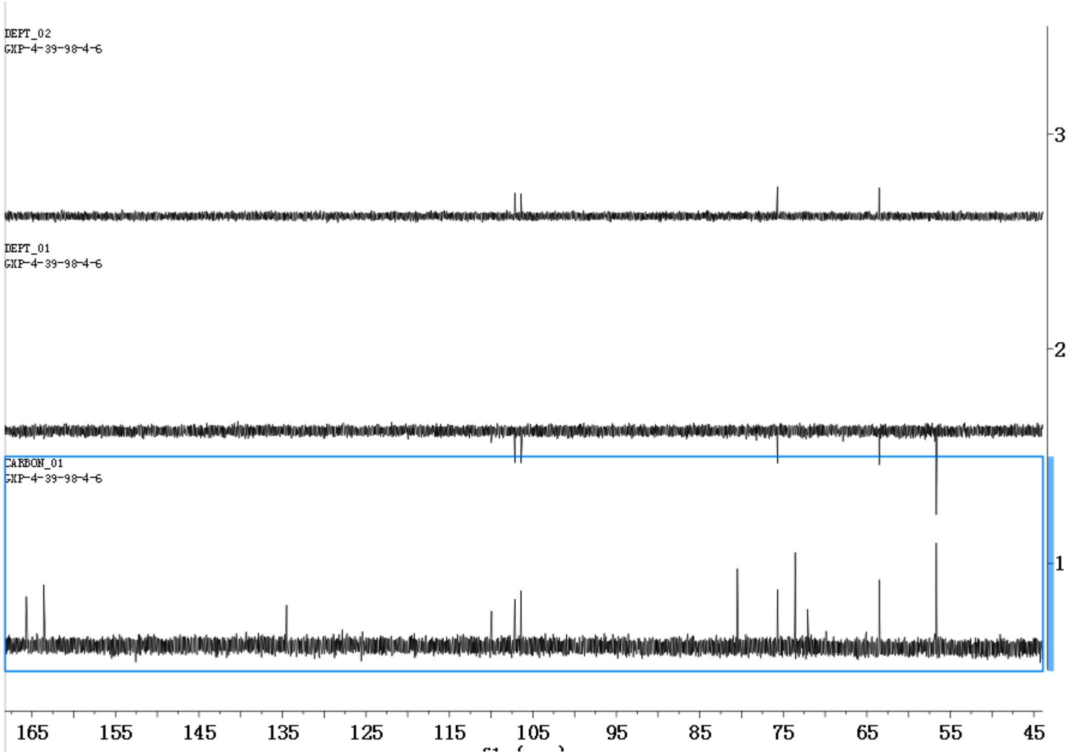

Figure S29. H-H COSY spectrum of auxarthrol G (**4**).

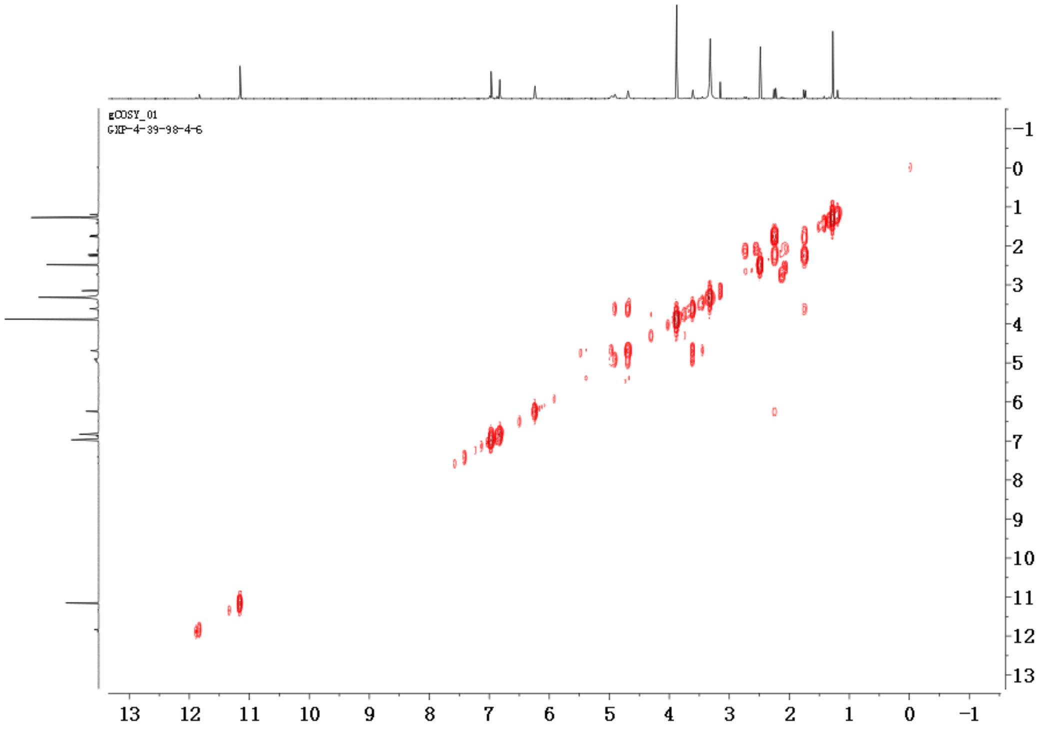

Figure S30. HSQC spectrum of auxarthrol G (**4**).

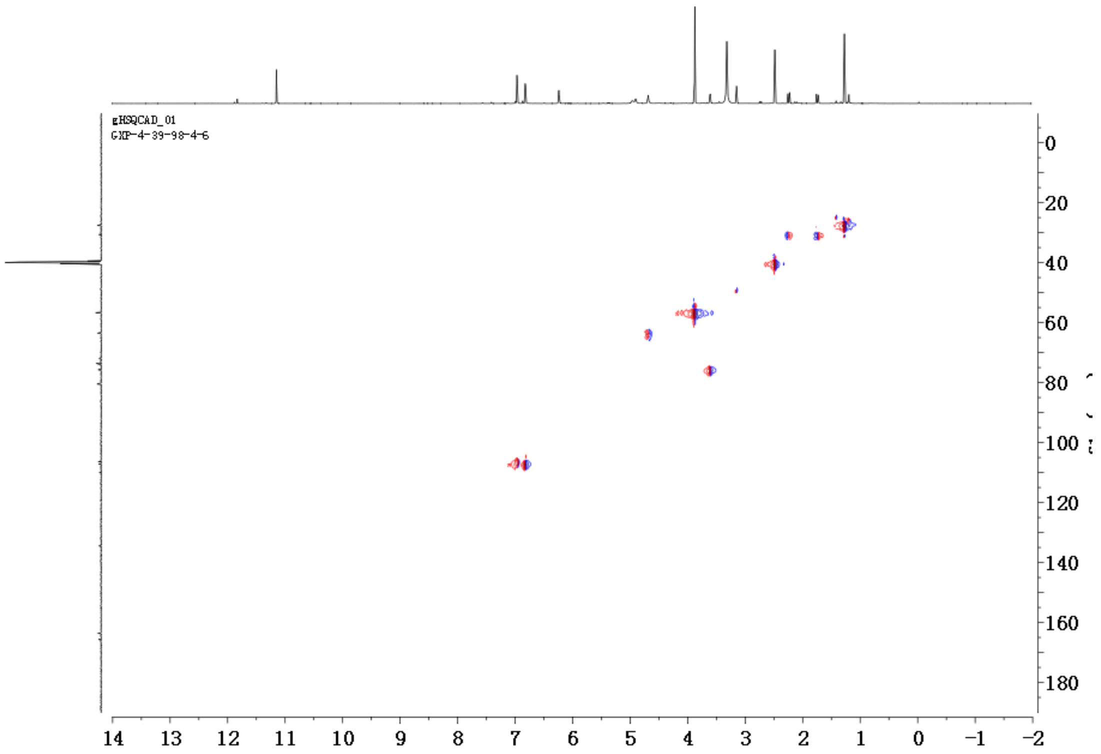

Figure S31. HMBC spectrum of auxarthrol G (**4**).

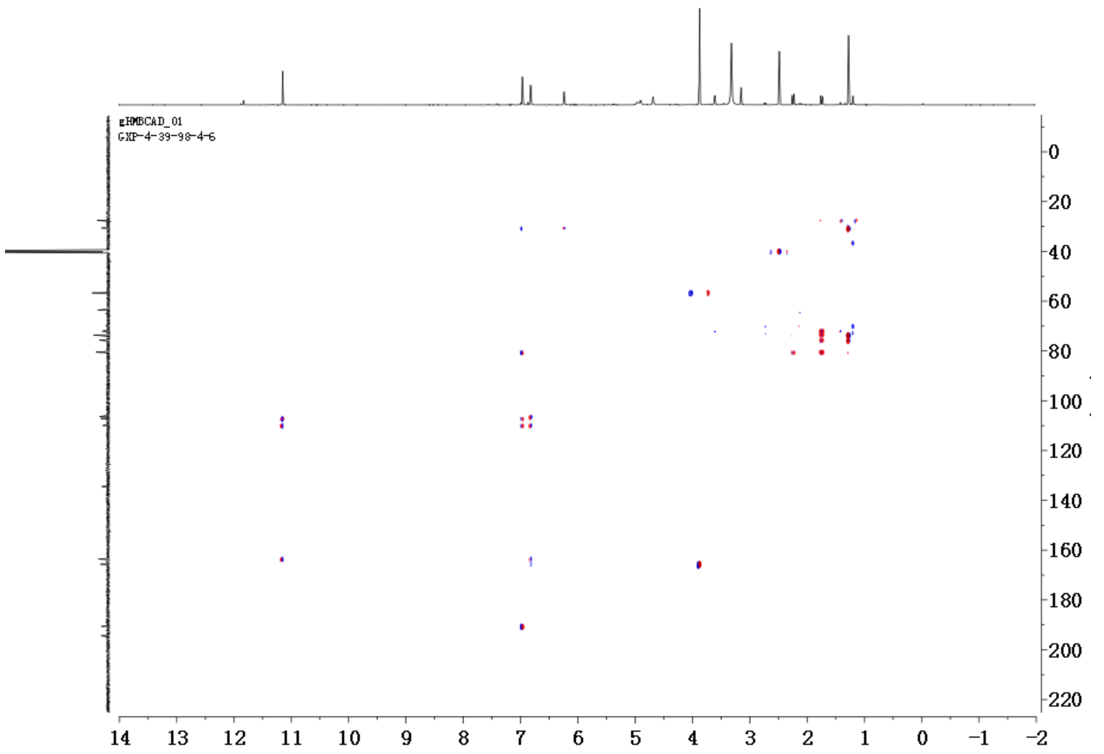

Figure S32. NOE spectrum of auxarthrol G (**4**).

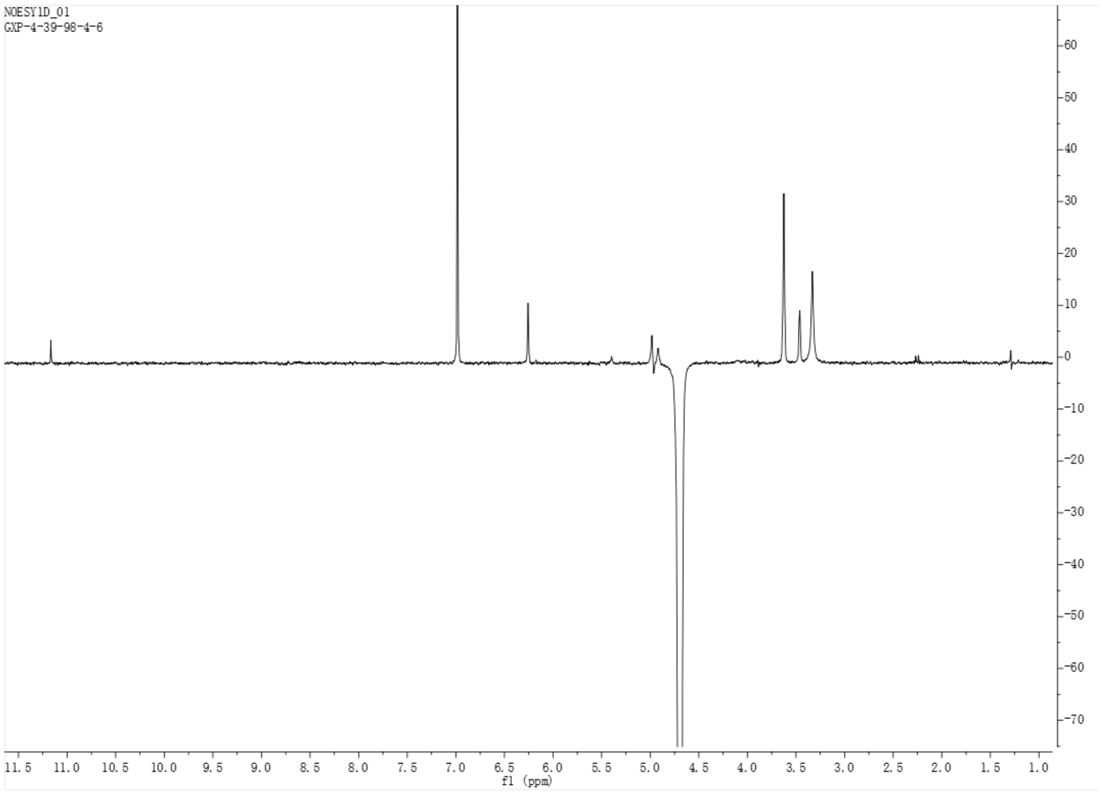

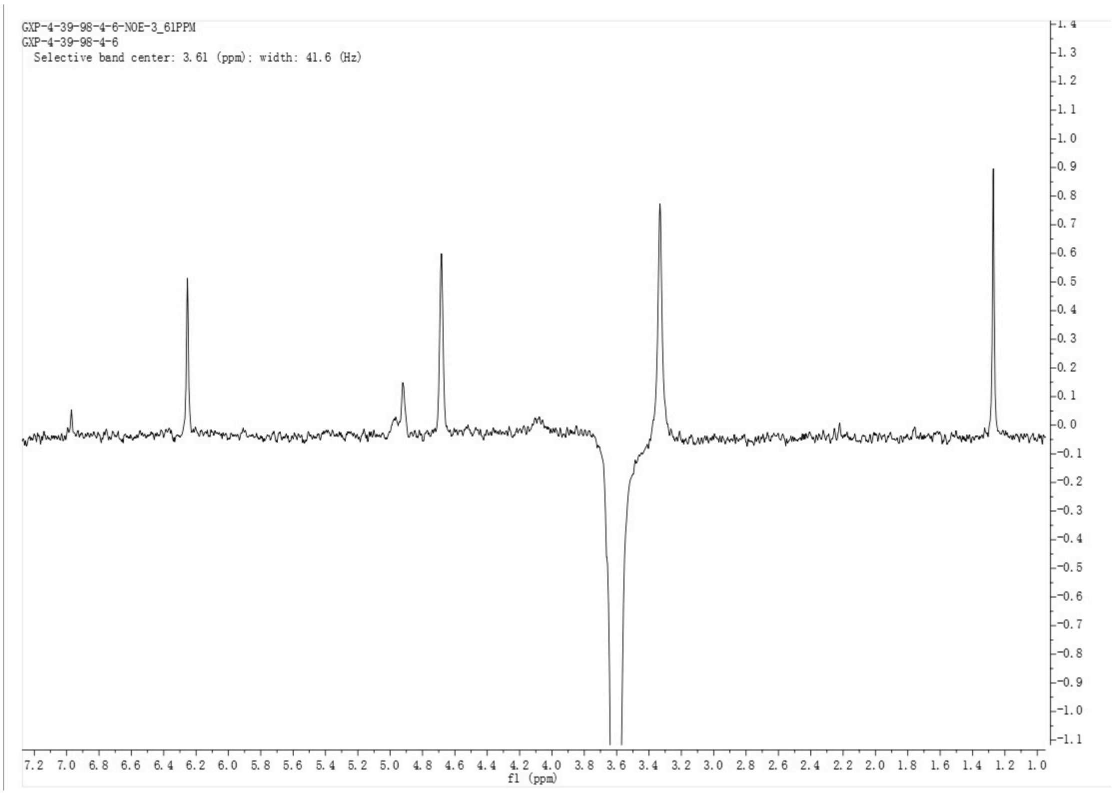

Figure S33. HRESIMS of auxarthrol G (**4**).

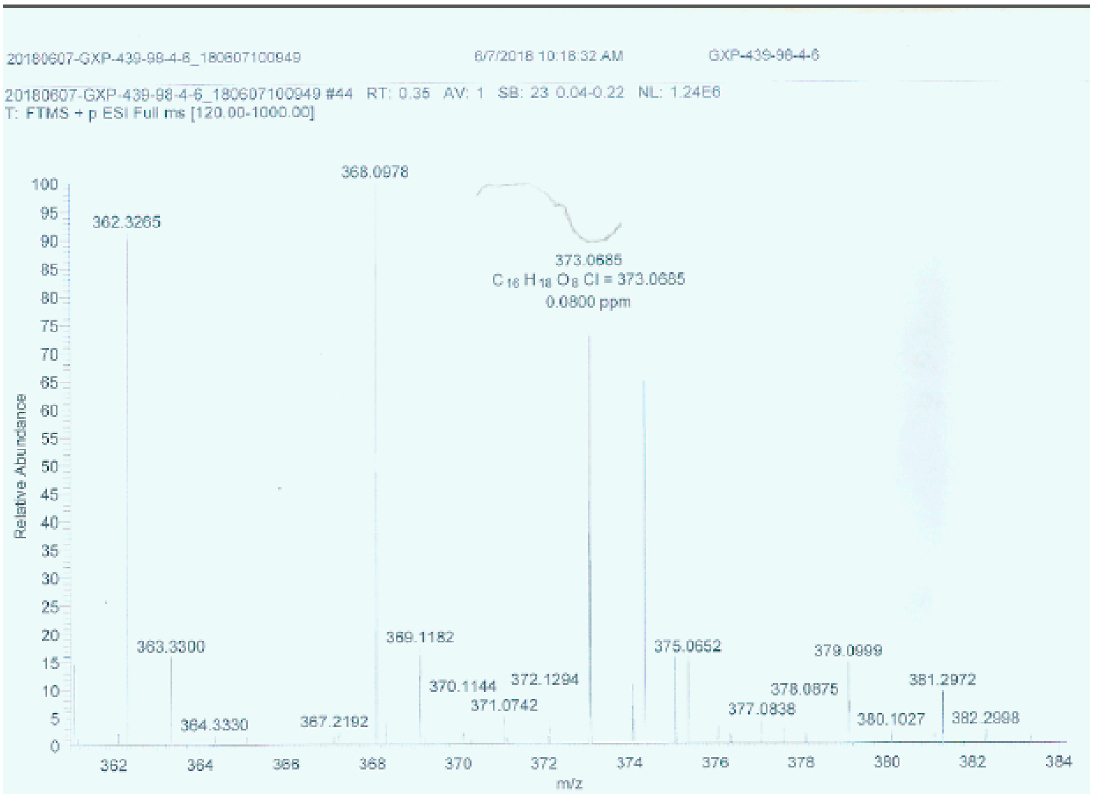

Figure 34. ^{1}H NMR spectrum (500 MHz) of auxarthrol H (**5**) in DMSO-d_6.

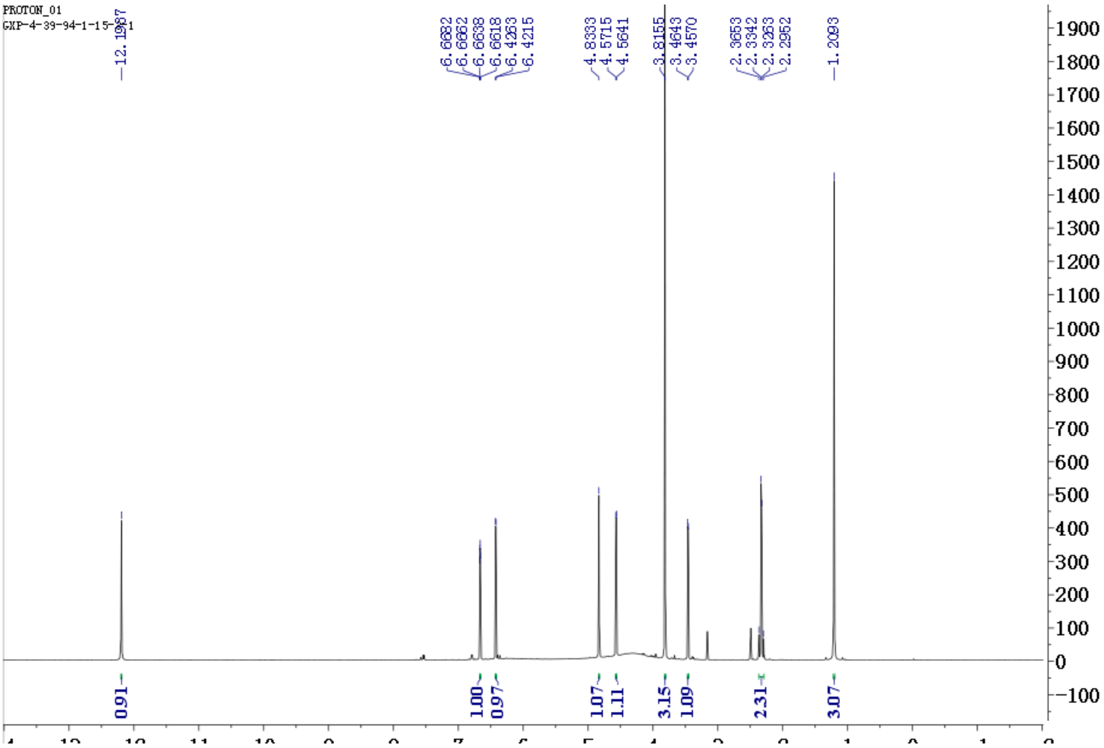

Figure 35. ^{13}C NMR spectrum (500 MHz) of auxarthrol H (**5**) in DMSO-d_6.

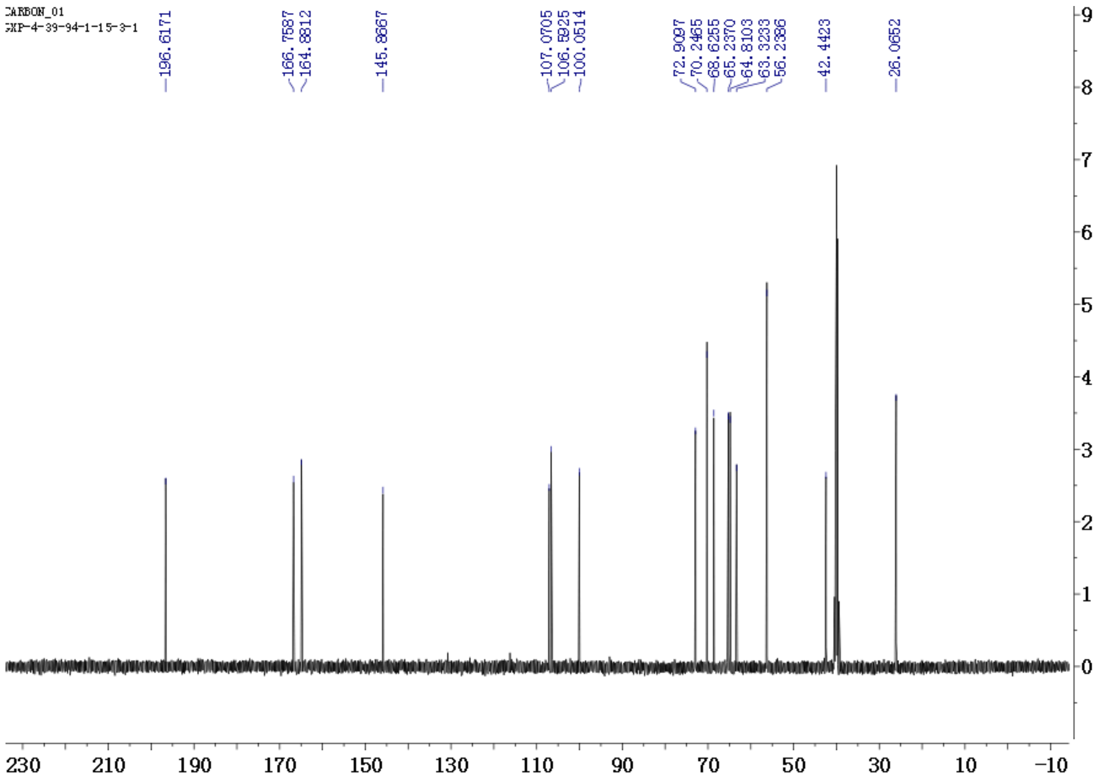

Figure 36. DEPT (125 MHz) spectrum of auxarthrol H (**5**).

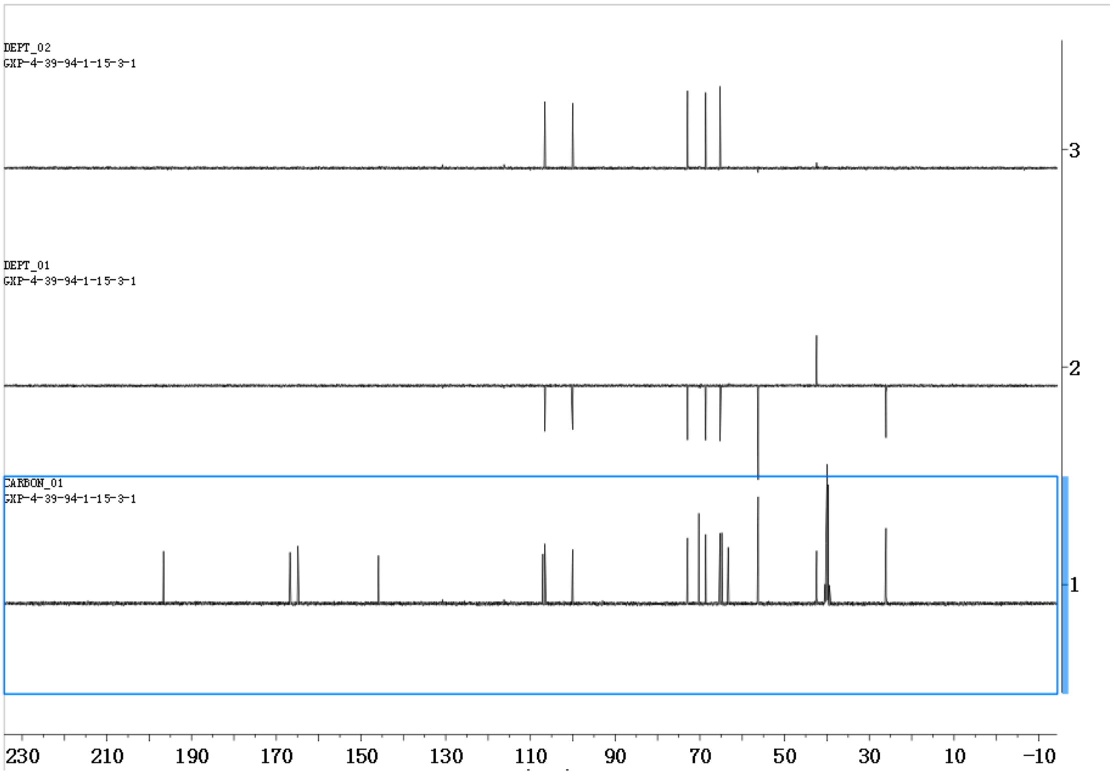

Figure 37. H-H COSY spectrum of auxarthrol H (**5**).

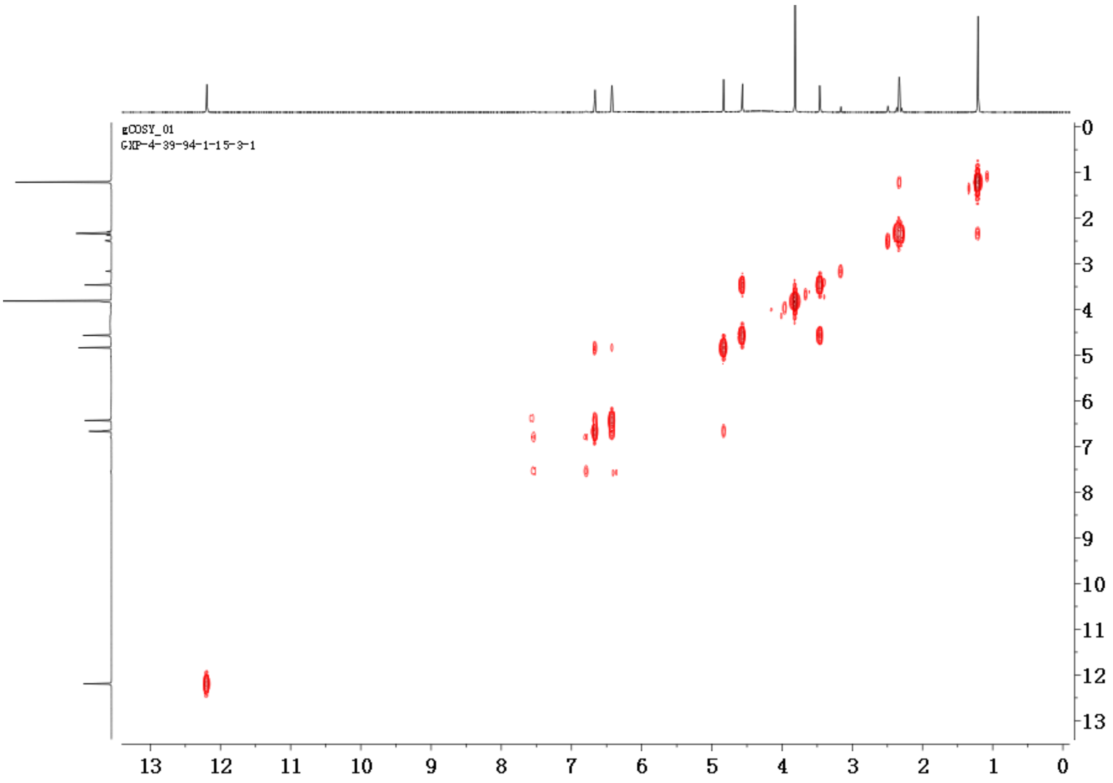

Figure 38. HSQC spectrum of auxarthrol H (**5**).

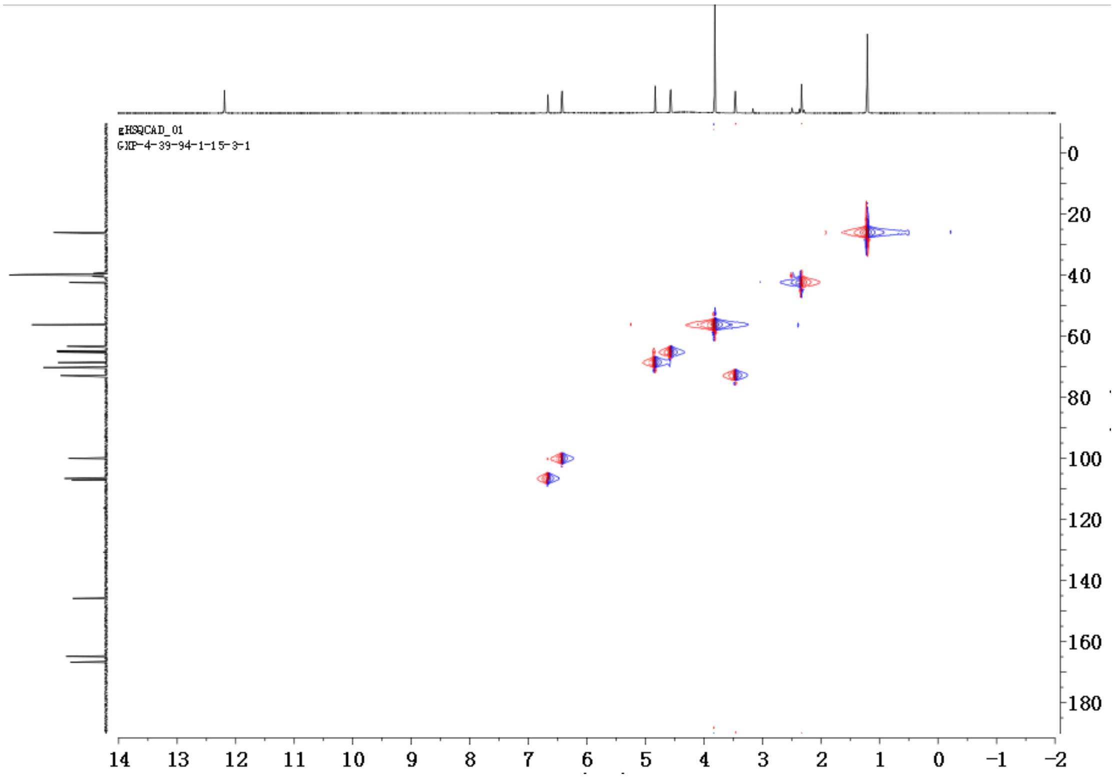

Figure 39. HMBC Spectrum of auxarthrol H (**5**).

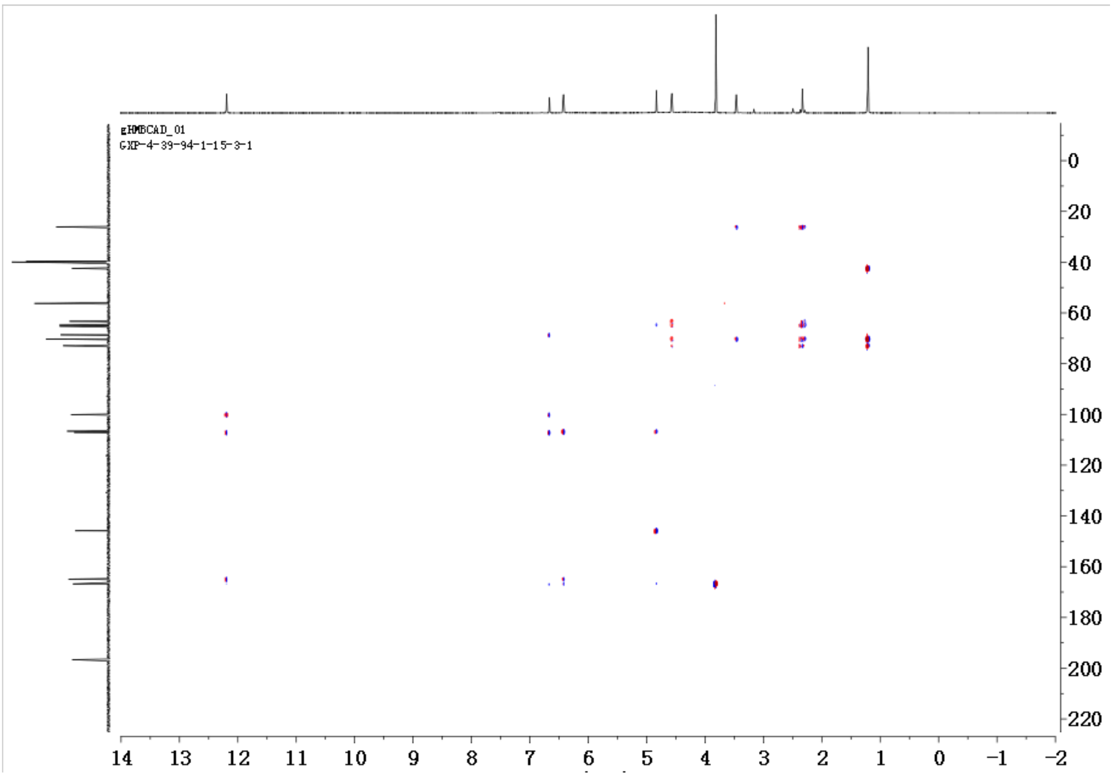

Figure 40. NOE spectrum of auxarthrol H (**5**).

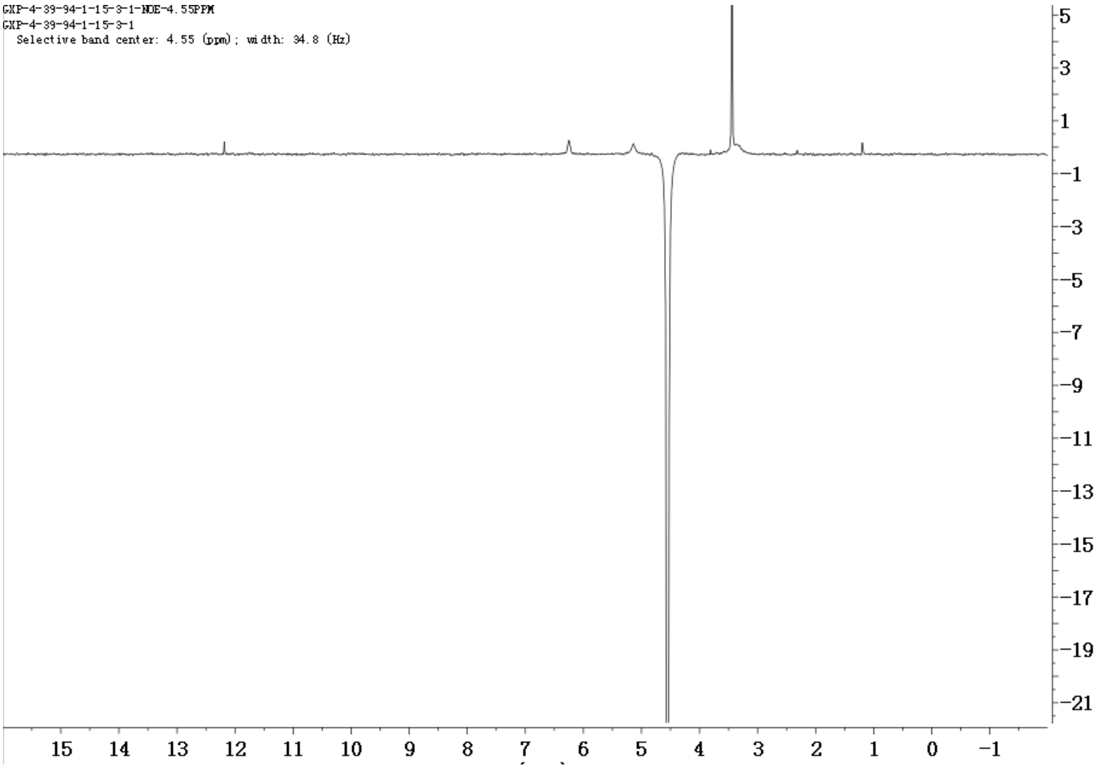

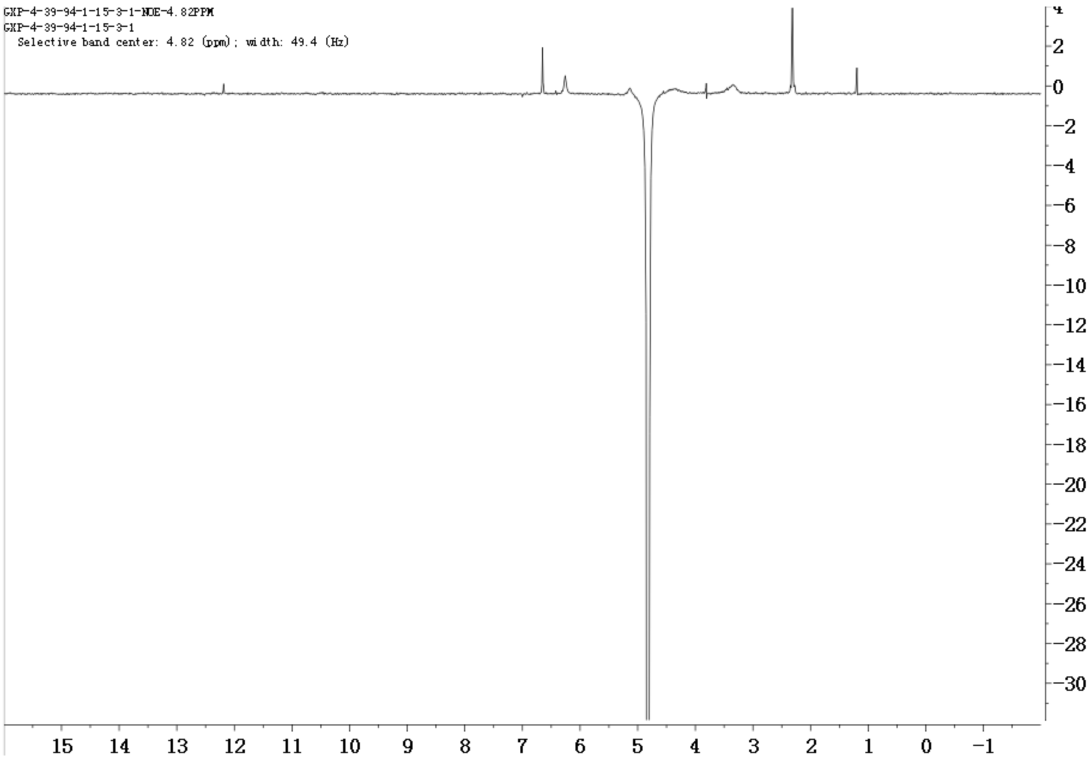

Figure 41. HRESIMS of auxarthrol H (**5**).

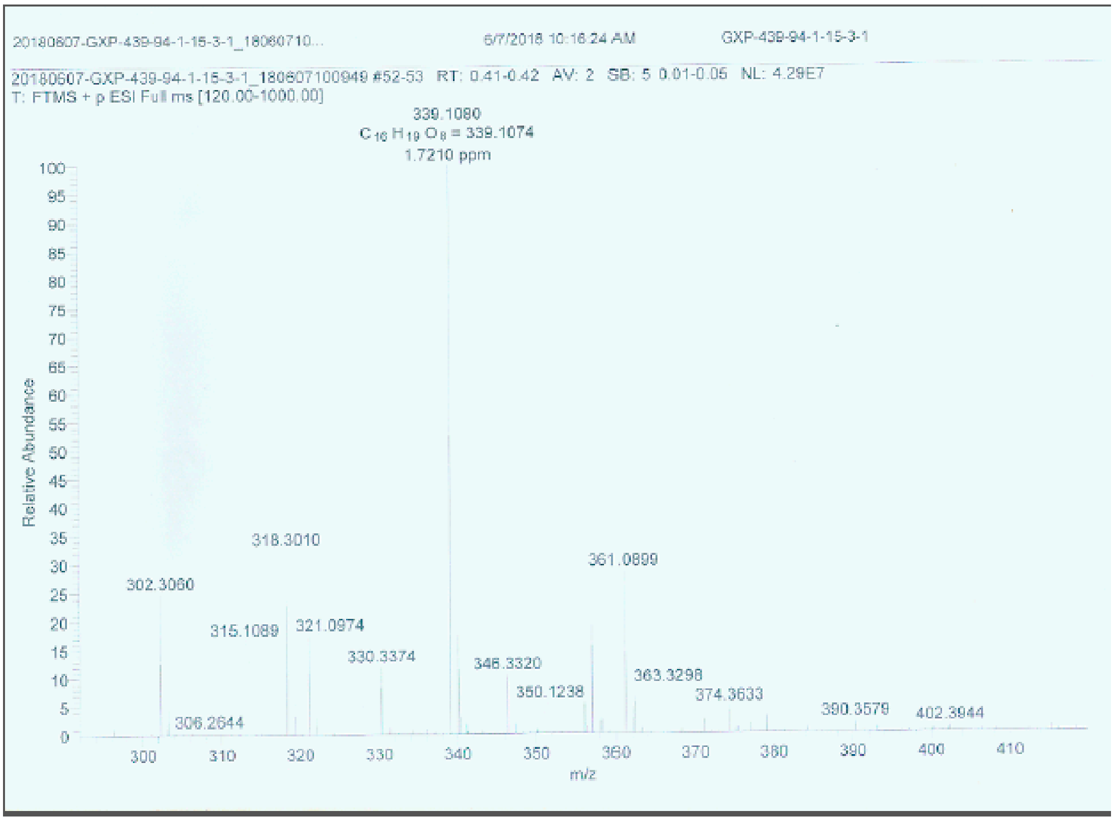

Supplement: Supplementary file 1 [file marinedrugs-17-00334-s001.pdf]
